# Destruction of Per- and Polyfluoroalkyl Substances in Reverse Osmosis Concentrate Using UV-Advanced Reduction Processes

**DOI:** 10.1021/acsestwater.4c00458

**Published:** 2024-10-28

**Authors:** Benjamin D. Fennell, Shawnee Chavez, Garrett McKay

**Affiliations:** † Zachry Department of Civil & Environmental Engineering Texas A&M University, College Station, Texas 77845, United States; ‡ Department of Civil & Environmental Engineering, University of Tennessee, Knoxville, Tennessee 37916, United States

**Keywords:** water treatment, PFAS, reduction, hydrated electron, sulfite

## Abstract

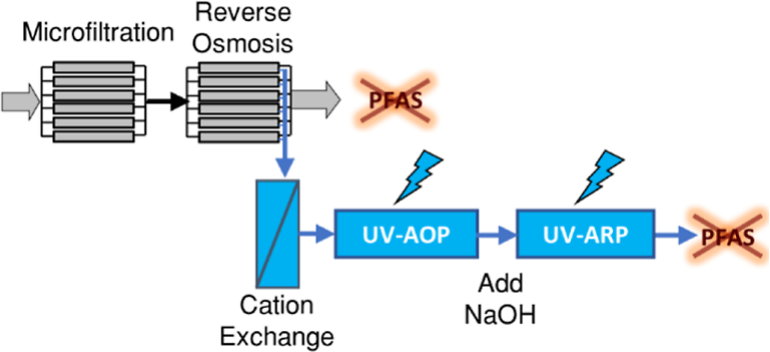

UV-advanced reduction processes (UV-ARP), characterized by the strongly reducing aqueous electron (e_aq_^–^), have been shown to degrade perfluoroalkyl and polyfluoroalkyl substances (PFAS). Due to the high cost of PFAS destruction technologies, concentrated waste streams derived from physical treatment processes, such as ion exchange or membrane concentrates, are promising targets for implementation of these technologies. However, there are limited studies on the application of UV-ARP for PFAS destruction in concentrated waste streams. This study evaluates the effectiveness of the UV/sulfite ARP in reverse osmosis concentrate (ROC) containing high concentrations of dissolved organic carbon (DOC), nitrate, and carbonate species, spiked with mg/L concentrations of perfluorooctanesulfonic acid, perfluorobutanesulfonic acid, perfluorooctanoic acid, and perfluorobutanoic acid. We demonstrate that hardness removal and preoxidation of ROC with UV/persulfate enables >90% PFAS defluorination within 24 h of subsequent UV/sulfite treatment, a 3-fold enhancement in defluorination % compared to UV/sulfite treatment without preoxidation. This enhancement is shown to result from abatement of the light shielding and e_aq_^–^ scavenging capacity of DOC during UV/persulfate oxidation. Collectively, these results demonstrate that appropriate pretreatment steps increase the effectiveness of PFAS destruction using UV-ARP, supporting the application of UV-ARP for PFAS destruction in ROC and other concentrated waste streams.

## Abstract 2

Reductive defluorination of per- and polyfluoroalkyl substances using the UV/sulfite advanced reduction process is evaluated in reverse osmosis concentrate. Results indicate that pretreatment of ROC with UV/persulfate results in a three-fold enhancement in the extent of defluorination achieved compared to UV/sulfite treatment alone.

## 1 Introduction

The production and use of per- and polyfluoroalkyl substances (PFAS) in consumer and industrial products have led to the widespread contamination of drinking water sources with ng L^–1^ to μg L^–1^ concentrations of PFAS.^1−7^ When ingested, PFAS are known to exhibit carcinogenic, reproductive, and immune effects.^8−11^ To address this concern, the United States Environmental Protection Agency finalized the National Primary Drinking Water Regulation (NPDWR) for six PFAS in April 2024.^12^ The regulation requires utility monitoring and reduction of the levels of these PFAS, potentially by implementing advanced treatment technologies, for public water systems that exceed the Maximum Contaminant Levels defined by the NPDWR.

Technologies currently used in the field to treat PFAS-contaminated drinking water sources include granular activated carbon, ion exchange, and membrane filtration. Despite their effectiveness in removing PFAS from water, these technologies also produce concentrated waste streams, which may contain hazardous substances under CERCLA [e.g., perfluorooctanoic acid (PFOA) and perfluorooctanesulfonic acid (PFOS)].^13^ Current methods for disposal of these waste streams risk the possibility of reintroducing PFAS into the environment. Therefore, technologies that quantitatively defluorinate C–F bonds in PFAS to fluoride ions (F^–^) are needed.^14−16^ Although many PFAS destruction technologies, such as plasma,^16,17^ electrochemical,^18−20^ hydrothermal,^21^ and UV-advanced reduction processes (UV-ARP),^22−26^ have been studied, the impact of water matrix constituents on their effectiveness has not been thoroughly investigated.

UV-ARP, in particular, has emerged as a promising PFAS destruction technology. PFAS destruction is accomplished in UV-ARP by the reduction of C–F bonds with aqueous electrons (e_aq_^–^), generated through the interaction of ultraviolet light (typically 254 nm) and electron donors (e.g., sulfite).^23,25^ Most UV-ARP studies have reported PFAS destruction in ultrapure water. For example, using the UV/SO_3_^2–^ + I^–^ system, Liu et al.^27^ reported EE/O values (a figure of merit for energy consumption) as low as 1.4 and 11.5 kW h m^–3^ for the destruction of PFOA and PFOS, respectively, in ultrapure water. These EE/O values are comparable to those from other PFAS destruction technologies applied in ultrapure water.^18^ Although some studies investigated the impact of water matrix constituents on PFAS defluorination,^28,29^ Fennell et al.^30^ was one of the first to directly quantify the impact of e_aq_^–^ concentration and scavenging capacity on UV-ARP destruction of PFAS. By simultaneously measuring PFOS destruction and fluence-normalized e_aq_^–^ exposure (*R*_e–,UV_) in the UV/SO_3_^2–^ system, Fennell et al.^30^ demonstrated that the long-term treatability of PFOS is less impacted by the initial e_aq_^–^ scavenging conditions, but it is rather influenced by the presence of e_aq_^–^ scavengers like dissolved organic carbon (DOC) and carbonate species. Likely related to the role of carbonate species as e_aq_^–^ scavengers, several recent studies have demonstrated increased PFAS destruction rates in the UV/sulfite system by increasing pH from 9.5 to 12.^31,32^ However, the effectiveness of this pH adjustment for degrading PFAS in concentrated waste streams with coexisting water matrix components has not been tested. In addition, the mechanism of enhanced PFAS destruction in the UV/sulfite system at pH 12 remains unclear. Although Bentel et al. hypothesized that the enhancement resulted from intrinsic differences in reactivity of C–F bonds at pH 12 vs pH 9.5,^31^ Amador et al. suggested that increased e_aq_^–^ scavenging by carbonate species at pH 9.5 was responsible.^32^ There are direct implications for this mechanism. For example, if C–F bonds or reaction intermediates are more reactive at pH 12, pH adjustment may be necessary regardless of the background matrix. On the other hand, if pH 12 renders e_aq_^–^ scavenging by carbonate species ineffective (e.g., due to highly reacting CO_2(aq)_),^32^ then other pretreatment options could be considered for removing alkalinity. Ultimately, the examples presented above highlight a few of the current knowledge gaps that limit the technology readiness of UV-ARP for PFAS treatment.

The twofold objectives of this study were to (i) evaluate the effectiveness of UV-ARP for the degradation of PFAS in reverse osmosis concentrate (ROC) and (ii) to provide recommendations for selecting the optimal initial concentration of e_aq_^–^ sensitizers in different treatment contexts (e.g., ultrapure water vs ROC). To address the first objective, we received ROC from an indirect potable reuse treatment train at the Orange County Water District (OCWD) Advanced Water Purification Facility. ROC was used in this study as a “real world water” test case because it contained high concentrations of DOC, nitrate, and alkalinity, which are expected to negatively impact UV-ARP performance. Four PFAS [PFOS, perfluorobutanesulfonic acid (PFBS), PFOA, and perfluorobutanoic acid (PFBA)] were spiked into the ROC prior to UV-ARP treatment at concentrations similar to PFAS concentrated waste streams (∼mg L^–1^). We demonstrate that ROC pretreatment including hardness removal (using cation exchange) and preoxidation via the UV/S_2_O_8_^2–^ system enables >90% defluorination within 24 h of subsequent UV/SO_3_^2–^ treatment, which is a 3-fold enhancement compared to optimized UV/SO_3_^2–^ conditions at pH 12 without UV/S_2_O_8_^2–^ preoxidation. This enhancement is hypothesized to result from abatement of the light shielding and e_aq_^–^ scavenging capacity of DOC during UV/S_2_O_8_^2–^. To address the second objective of this study, we used the *R*_e^–^,UV_ method to probe the mechanism of enhanced PFAS degradation reported at pH 12 in the UV/SO_3_^2–^ system in ultrapure water containing carbonate at pH 10 and 12. Collectively, these results demonstrate that ROC pretreatment increases the effectiveness of PFAS destruction using UV-ARP, supporting the application of UV-ARP for PFAS destruction in ROC and other concentrated waste streams.

## 2 Materials and Methods

Details of the chemicals and experimental procedures are described in the Supporting Information Text S1, including chemicals and solution preparation, characterization of ROC, hardness removal pretreatment, analytical methods, and calculation of *R*_e_^–^_,UV_.

### 2.1 UV/S_2_O_8_^2–^Pretreatment

UV/S_2_O_8_^2–^ pretreatment of ROC was conducted in duplicate immersion well reactors (Ace Glass) with an exterior glass body and an interior quartz sleeve. Prior to irradiation, a 10 W low-pressure Hg lamp emitting at 254 nm (Norman Lamps GPH212T5*L*/4P) was placed into the immersion sleeve and powered on for 15 min with no sample in the reactors. UV irradiance was measured monthly using uridine actinometry^33^ with values ranging between 0.99 × 10^–8^ and 1.21 × 10^–8^ Es cm^–2^ s^–1^ (Text S1.4). The average reactor path length was determined as 2.23 ± 0.02 cm.^30^ The temperature of each reactor was controlled at 20 °C using a recirculating chiller, while a magnetic stirring bar continuously stirred at 400 rpm. A mixed solution of ROC, 25.0 mM potassium persulfate, and PFAS was added for a total solution volume of 541.5 mL. The PFAS mixture included PFOS, PFBS, PFOA, and PFBA, each at 25 μM. To evaluate the formation of quantifiable PFAS from precursors native to the ROC during oxidation treatment, one control experiment included a mixed solution of ROC and 25.0 mM potassium persulfate without the spiked PFAS mixture. The solution was mixed in the reactor for at least 30 s before the lamps were turned on. Aliquots (3 mL) were sampled at predetermined time intervals using a stainless-steel needle and glass syringe. PFAS samples were stored in Falcon tubes at 4 °C prior to analysis (3 months or less). Cation and anion samples were collected in 1.5 mL polypropylene vials and analyzed immediately.

### 2.2 UV-ARP Treatment

UV-ARP experiments were conducted in duplicate under similar experimental conditions as described above for UV/S_2_O_8_^2–^ experiments, including 15 min of warm up time unless the lamp was previously powered on. A 570 mL solution was prepared in either ultrapure water or ROC containing monochloroacetic acid (MCAA) (e_aq_^–^ probe compound), sodium sulfite or indole acetic acid (IAA) (e_aq_^–^ sensitizers), and PFAS mixture. A MCAA concentration of 50 μM was used in this study to improve detection in the ROC matrix (e_aq_^–^ scavenging by MCAA is minimal in the ROC matrix). Experiments in ultrapure water contained either 1.0 mM borate buffer (pH 10) or 10 mM carbonate buffer (pH 10 or 12). Nitrogen gas was bubbled 45 min prior to starting each experiment, and bubbling was maintained during irradiation for experiments done in ultrapure water (but not ROC). Samples for ion chromatography and PFAS analysis were collected as described in Supporting Information Text S1.4, and aliquots for sulfite analysis were collected in 20 mL borosilicate glass scintillation vials.

### 2.3 ROC Experimental Matrix

Several experiments were attempted to maximize the rates of PFAS destruction in the ROC (Table 1). ROC was first pretreated with cation exchange resin to remove water hardness (Text S1.3). UV/SO_3_^2–^ experiments were conducted at pH 12 based on the faster rates of PFAS destruction reported in prior literature^31^ and preliminary experiments showing <30% defluorination in the UV/SO_3_^2–^ system at pH 10 in ROC. The UV/S_2_O_8_^2–^ + UV/SO_3_^2–^ experiment was maintained at pH ∼ 12 by dosage of NaOH during experiments to favor the formation of ^•^OH as the oxidant. pH was not controlled in the UV/S_2_O_8_^2–^ + UV/SO_3_^2–^ spike experiment, so both ^•^OH and SO_4_^•–^ were generated in this treatment (the final pH was ∼5). In the UV/S_2_O_8_^2–^ + UV/SO_3_^2–^ spikes experiment, we observed a more rapid decrease in the sulfite concentration during subsequent UV/SO_3_^2–^ treatment, which is attributed to quenching by residual persulfate not consumed during UV/S_2_O_8_^2–^ (see Table S7). Therefore, additional sulfite was spiked at 12 and 24 h to maintain an adequate concentration of e_aq_^–^ for PFAS destruction.

**Table 1:** Experimental Matrix for PFAS Destruction in ROC

label	description	rationale
UV/SO_3_^2–^	ROC was spiked with PFAS mixture (25 μM), adjusted to pH 12, and dosed with [SO_3_^2–^] = 50 mM	pH 12 has been shown in ultrapure water to enhance PFAS degradation rates in the UV/SO_3_^2–^ system. Sulfite concentration chosen based on the model presented in Text S2
UV/IAA	ROC was spiked with PFAS mixture (25 μM), adjusted to pH 12, and dosed with [IAA^–^]_0_ = 2.5 mM	IAA has a higher molar absorption coefficient at 254 nm and higher e_aq_^–^ quantum yield. IAA concentration chosen based on the model presented in Text S2
UV/S_2_O_8_^2–^ + UV/SO_3_^2–^	ROC was spiked with PFAS mixture (25 μM), dosed with 25 mM K_2_S_2_O_8_, and irradiated for 1.25 h. A pH ∼ 12 was maintained by adding 1 g of solid NaOH during UV/S_2_O_8_^2–^. After oxidation, the mixture was dosed with sulfite to quench remaining persulfate, then dosed with additional sulfite such that [SO_3_^2–^] = 50 mM at the beginning of reductive treatment	maintaining a pH ∼ 12 favors the formation of ^•^OH in the UV/S_2_O_8_^2–^ system. Oxidation of DOM chromophores is expected to enhance the rate of reductive treatment by UV/SO_3_^2–^
UV/S_2_O_8_^2–^ + UV/SO_3_^2–^ spikes	ROC was spiked with PFAS mixture (25 μM), initially adjusted to pH 8.3, dosed with 25 mM K_2_S_2_O_8_ and irradiated for 1.25 h. After oxidation, the solution pH was ∼5. The mixture was adjusted to pH 12, then dosed with additional sulfite such that [SO_3_^2–^] = 50 mM at the beginning of reductive treatment. Additional sulfite was dosed at 12 h ([SO_3_^2–^] = 25.6 mM) and 24 h ([SO_3_^2–^] = 14.6 mM)	the decrease in pH during UV/S_2_O_8_^2–^ implies that both ^•^OH and SO_4_^•–^ were formed in the UV/S_2_O_8_^2–^ system. Oxidation of DOM chromophores is expected to enhance the rate of reductive treatment by UV/SO_3_^2–^

The UV/S_2_O_8_^2–^ + UV/SO_3_^2–^ experiment also included an additional 1 h treatment time prior to UV/SO_3_^2–^ treatment, whereas other experiments did not. During this 1 h, 60 mM sulfite was added to each reactor and allowed to react under dark conditions (i.e., no UV light) to quench any remaining persulfate. Initial UV-ARP sensitizer concentrations were selected as 50 mM sulfite and 2.5 mM IAA based on the results presented in Text S2. Sulfite was selected as a sensitizer due to its extensive use in previous studies.^34−36^ IAA was selected due to its high molar absorption coefficient and use as an e_aq_^–^ sensitizer in previous studies.^37−40^

### 2.4 Metrics Used to Compare PFAS Destruction

Several metrics were used to compare PFAS destruction. Since the same wattage lamps were used for all experiments, the defluorination % can be directly compared across different treatment scenarios. *R*_e^–^,UV_ was used to compare the amount of e_aq_^–^ available for PFAS destruction between different treatments. To compare PFAS destruction between UV-ARP and other treatment technologies, we calculated both EE/O and EE/Max.deF. The EE/O (kW h m^–3^) represents the electrical energy required to decrease the concentration of a contaminant by 1 order of magnitude in a unit volume of water.^41^ While EE/O has been widely used for comparing PFAS treatment technologies, it only accounts for loss of the parent compound. Alternatively, Liu and co-workers introduced EE/Max.deF,^27^ which represents the electrical energy required to reach 90% of the maximum defluorination.

## 3 Results and Discussion

### 3.1 ROC Water Quality Parameters

The ROC was characterized by high concentrations of DOC (51.4 mg_C_ L^–1^), nitrate (40.3 mg-N L^–1^), and alkalinity (1350 mg L^–1^ as CaCO_3_), as shown in Table 2. The *A*_254_ was 0.964 cm^–1^ (10.9% transmittance). PFAS were present at ng L^–1^ levels, including 6:2 fluorotelomer sulfonic acid (6:2 FTS), perfluorohexanoic acid (PFHxA), and perfluoropentanoic acid (PFPeA) (Table 2). These background PFAS levels in the ROC are not expected to impact the interpretation of UV-ARP experiments. Precipitate formation was observed during initial attempts to increase the pH to 12 (Figure S2), which was presumed to contain calcium carbonate and magnesium salts given the decrease in concentration of these cations after hardness removal (Table 2) and observed effervescence upon addition of concentrated HCl to the precipitate. To avoid light screening caused by suspended particles, ROC was passed through cation exchange resin (hardness <50 mg L^–1^ as CaCO_3_ after pretreatment), a strategy employed also in prior literature for treating PFAS-contaminated groundwater.^15^ PFAS concentrations in the ROC were higher after cation exchange. This is likely attributable to analytical variability due to our SPE protocol that was optimized for cleanup of higher concentrations of PFAS, not sample concentration.

**Table 2:** Water Quality Parameters for Native ROC, ROC Passed through Cation Exchange Resin, and ROC Preoxidized with UV/S_2_O_8_^2–^

parameter	ROC	ROC with cation exchange	ROC with cation exchange after 1.25 h UV/S_2_O_8_^2–^ Pretreatment
General Water Quality Parameters			
DOC (mg_C_ L^–1^)	51.4	52.1	11.0
pH	7.8	8.3	4.7
*A*_254_ (cm^–1^)	0.964	0.966	0.348
specific conductance (μS cm^–1^)	9760	10,400	14,360
alkalinity (mg L^–1^ as CaCO_3_)	1350	1340	78.0
Inorganic Ions			
nitrate (mg N L^–1^)	40.4	40.5	55.9
nitrite (mg N L^–1^)	7.8	7.9	LOQ^a^
ammonium (mg N L^–1^)	14.2	13.0	LOQ
fluoride (mg L^–1^)	5.2	5.1	5.9
bromide (mg L^–1^)	6.7	7.3	2.3
chloride (mg L^–1^)	3900	3600	1710
sodium (mg L^–1^)	1970	3460	3220
potassium (mg L^–1^)	154	22.0	2030
magnesium (mg L^–1^ as CaCO_3_)	1040	49.1	61.2
calcium (mg L^–1^ as CaCO_3_)	1250	34.8	51.9
PFAS, Bromate, and Chlorate			
6:2 FTS (ng L^–1^)	81.0	165	105
PFHxA (ng L^–1^)	189	932	668
PFPeA (ng L^–1^)	50.2	208	211
PFOA (ng L^–1^)	LOQ	77.2	155
PFOS (ng L^–1^)	LOQ	LOQ	88.7
PFBA (ng L^–1^)	LOQ	LOQ	93.0
PFBS (ng L^–1^)	LOQ	LOQ	135
bromate (mg L^–1^)	LOQ	LOQ	2.5
chlorate (μg L^–1^)	51.8	59.6	252

a Limit of quantitation: species (LOQ): nitrite (0.1 mg L^–1^), ammonium (0.1 mg L^–1^), PFAS (between 0.05 and 0.25 μg L^–1^), bromate (0.1 mg L^–1^), chlorate (0.05 mg L^–1^).

### 3.2 Selecting the Optimal e_aq_^–^ Sensitizer Concentration

Based on the high *A*_254_ of the ROC, it was anticipated that a higher concentration of e_aq_^–^ sensitizer would be required to achieve sufficient e_aq_^–^ formation. Prior research has shown minimal enhancements in defluorination at sulfite concentrations above 20 mM, but these results were from UV-ARP experiments performed in ultrapure water.^42^ In addition, because species like sulfite and IAA can act as both e_aq_^–^ sources and sinks, there is inherently some trade off in increasing the e_aq_^–^ sensitizer concentration. To determine the optimal sensitizer concentration, we applied a model to calculate [e_aq_^–^] that considers the background water absorbance, e_aq_^–^ scavenging capacity, e_aq_^–^ quantum yield, and the bimolecular rate constant between e_aq_^–^ and the sensitizer (see Supporting Information eq S7, Texts S2.1 and S2.4).

Employing this model resulted in optimal sensitizer concentrations that are dependent on the source water quality. For ultrapure water, the optimal [SO_3_^2–^] was 10 mM and the optimal [IAA] was 75 μM. Modeled and measured [e_aq_^–^] were within a factor of 2 (Figure S5), with the differences attributable to uncertainties in the e_aq_^–^ bimolecular rate constants for these species. Notably, IAA scavenges e_aq_^–^ at relatively low concentrations due to its high e_aq_^–^ reaction rate constant (∼10^8^ M^–1^ s^–1^),^43^ which may explain why a prior study did not observe any difference in the defluorination % of PFOA photolysis in the presence and absence of 1 mM IAA.^38^

**Figure 1:**
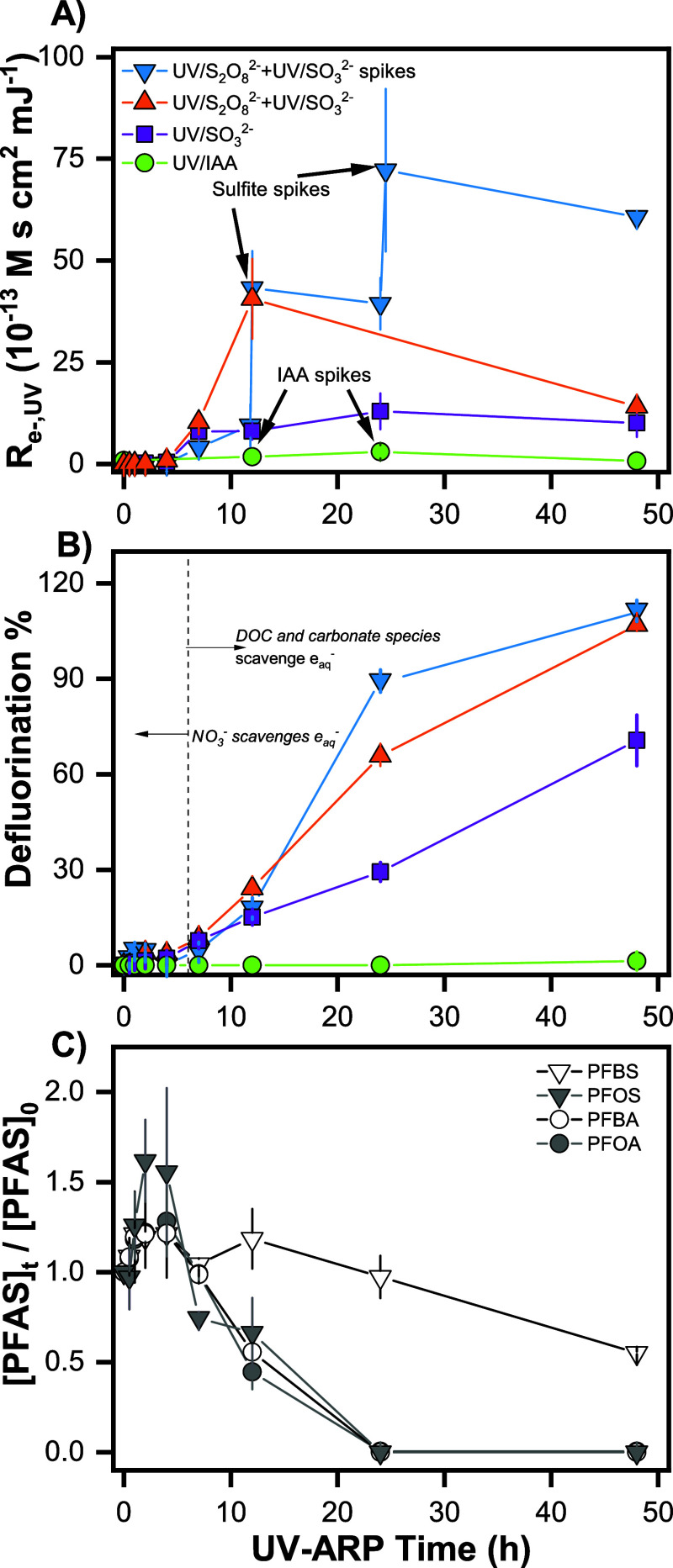
Photochemical treatment of PFAS spiked into ROC under four conditions: (1) UV/IAA, (2) UV/SO_3_^2–^, (3) UV/S_2_O_8_^2–^ + UV/SO_3_^2–^, and (4) UV/S_2_O_8_^2–^ + UV/SO_3_^2–^ spikes. *R*_e^–^,UV_ results and defluorination % for all treatment scenarios are shown in (A,B), respectively. Legend in (A) applies to (B). Degradation of parent PFAS from the UV/S_2_O_8_^2–^ + UV/SO_3_^2–^ spikes treatment is shown in (C). Markers represent the mean of duplicate measurements, and error bars represent the range between the duplicates (some error bars are within markers). Experimental conditions for each of the four treatment scenarios are described in Table 1. General conditions: 10 W low-pressure Hg lamp, pH_0_ = 9.5 or 12, 20 °C, [S_2_O_8_^2–^]_0_ = 25.0 mM, [SO_3_^2–^]_0_ = 50 mM, and [IAA]_0_ = 2.5 mM.

The model predicted that the optimal concentrations of IAA (2.5 mM) and sulfite (50 mM) would be higher in ROC than in ultrapure water. As for ultrapure water, [e_aq_^–^] measured in the ROC at the optimal sensitizer concentrations agreed with model predictions within a factor of 2. The initial [e_aq_^–^] produced by IAA and sulfite (∼10^–14^ M) are about 1000-fold lower than the initial [e_aq_^–^] measured in ultrapure water (Figure S5), consistent with the low UV transmission and high concentration of e_aq_^–^ scavengers present in the ROC. Despite this initially low [e_aq_^–^], *R*_e^–^,UV_ measured in the UV/S_2_O_8_^2–^ + UV/SO_3_^2–^ spikes treatment after 12 h (∼4 × 10^–12^ M s cm^2^ mJ^–1^, Figure 1A) was comparable to initial *R*_e^–^,UV_ values we reported previously in ultrapure water at pH 9.5 (∼5 × 10^–12^ M s cm^2^ mJ^–1^).^30^ In contrast, the maximum *R*_e^–^,UV_ in the UV/IAA system employed in ROC was lower by an order of magnitude (∼3 × 10^–13^ M s cm^2^ mJ^–1^, Figure S18). Results from experiments performed with IAA and sulfite demonstrate the benefits of employing sulfite as an e_aq_^–^ source even if IAA has a higher light absorption at the actinic wavelength. We expect that the model developed for optimizing sensitizer concentration will be useful in future UV-ARP research.

### 3.3 UV/S_2_O_8_^2–^ Pretreatment Followed by UV/SO_3_^2–^ Maximizes PFAS Destruction in ROC

**Figure 2:**
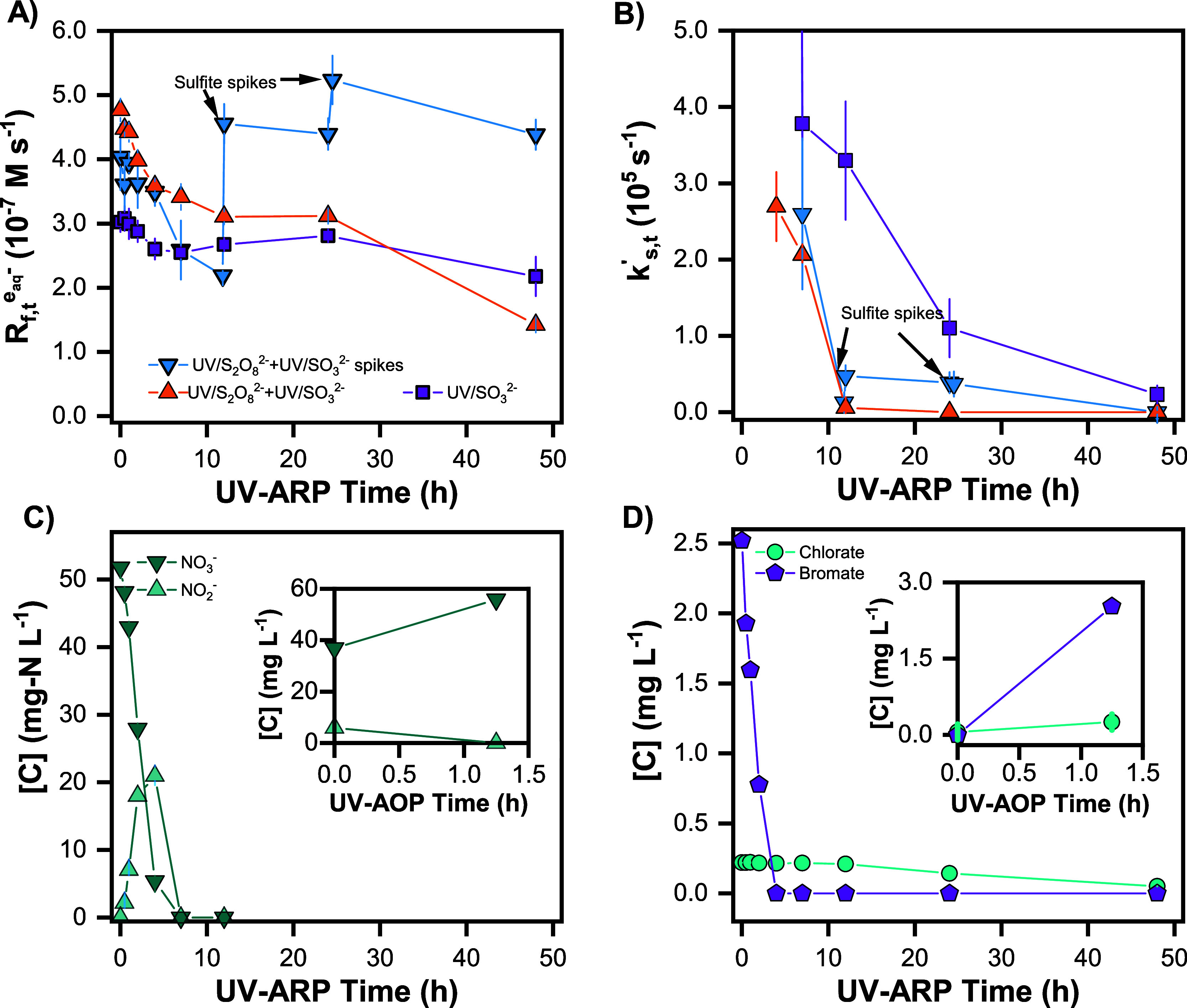
Time dependence of (A) rate of e_aq_^–^ formation () and (B) e_aq_^–^ scavenging capacity (*k*_S,*t*_^′^) during UV/SO_3_^2–^ treatment in ROC. Legend in (A) also applies to (B). (C,D) show time profiles of co-occurring contaminants for the UV/S_2_O_8_^2–^ + UV/SO_3_^2–^ spikes treatment both during the oxidation stage (insets) and reductive treatment. Markers represent the mean of duplicate measurements, and error bars represent the range between the duplicates (some error bars are within markers). Experimental conditions for each of the four treatment scenarios are described in Table 1. General conditions: 10 W low-pressure Hg lamp, pH_0_ = 9.5 or 12, 20 °C, [S_2_O_8_^2–^]_0_ = 25.0 mM, and [SO_3_^2–^]_0_ = 50 mM.

Of the four scenarios tested, the UV/S_2_O_8_^2–^ + UV/SO_3_^2–^ spikes treatment resulted in the greatest parent PFAS attenuation, defluorination %, and abatement of oxyanions (Figures 1 and 2). This treatment also had the largest cumulative *R*_e^–^,UV_ (Figure 1A). In comparison, UV/S_2_O_8_^2–^ + UV/SO_3_^2–^ (without sulfite spikes) had a lower defluorination % at 24 h (∼70%, Figure 1B) but a similar defluorination % at 48 h. Experiments employing the optimized UV/SO_3_^2–^ conditions from prior literature (i.e., pH 12) without preoxidation showed a significantly reduced *R*_e^–^,UV_, resulting in a defluorination of <30% at 24 h (Figures 1A,B and S17). In the UV/IAA system, minimal parent PFAS attenuation (<20%) and defluorination (<1%) were observed (Figure S18). This result was surprising because experiments in ultrapure water indicated that IAA and sulfite photolysis produced similar [e_aq_^–^] (∼10^–12^ to 10^–11^ M, see Figure S5). This finding indicates that although IAA has a high molar absorption coefficient and quantum yield of e_aq_^–^ formation, it is ineffective for PFAS destruction via UV-ARP in ROC and potentially other complex water matrices (see Text S3.1 for additional discussion).

UV/S_2_O_8_^2–^ pretreatment of ROC led to a 3-fold improvement in the extent of PFAS destruction after 24 h of subsequent UV/SO_3_^2–^ treatment compared to no preoxidation. The *R*_e^–^,UV_ values, parent compound attenuation, and defluorination % observed in this maximized system even exceeds that in the UV/SO_3_^2–^ system at pH 9.5 in ultrapure water.^30,36,42^ To compare the maximized UV-ARP system to those of other studies, we calculated two metrics that incorporate energy usage. First, EE/O for the individual PFAS were calculated based on the kinetic data shown in Figure 1C. EE/O values for PFOS, PFBA, and PFOA in ROC preoxidized with UV/S_2_O_8_^2–^ have an upper limit of 370 kW h m^–3^ based on the kinetic data in Figure 1C. Importantly, this value includes the time required for both UV/S_2_O_8_^2–^ (∼1 h) and UV/SO_3_^2–^ treatment (24 h). This EE/O is approximately 4-fold larger than the 89 kW h m^–3^ for PFOS destruction in ultrapure water in the pH 12 UV/SO_3_^2–^ system at 254 nm irradiation.^27^ The near equivalent EE/O values for PFOS, PFBA, and PFOA are consistent with the similar reactivity of perfluoroalkyl carboxylic acids (PFCAs) and perfluorosulfonic acids (PFSAs), as previously reported at pH 12.^31,44^ EE/Max.deF was also calculated for the PFAS mixture (Figure 1B). For the UV/S_2_O_8_^2–^ + UV/SO_3_^2–^ spikes treatment, EE/Max.deF was 440 kW h m^–3^, which is less than a factor of 2 greater than the EE/Max.deF reported by Liu et al. (∼240 kW h m^–3^) for the pH 12 UV/sulfite system at 254 nm irradiation in ultrapure water.^27^ Collectively, these results demonstrate that sample pretreatment, in this case, oxidation of DOC with UV/S_2_O_8_^2–^, can decrease the energy usage during subsequent reductive treatment to values comparable with UV-ARP applied in ultrapure water.

When considering metrics for energy usage, it should be noted that typical energy requirements for filtration technologies such as nanofiltration and reverse osmosis (<5 kW h m^–3^)^15^ are much less than the EE/O and EE/Max.deF observed here. Thus, PFAS destruction is expected to be the main contributor to energy usage in membrane-based concentrate-then-destroy technologies.

### 3.4 Comparison of *R*_e^–^,UV_, e_aq_^–^ Formation Rates, and e_aq_^–^ Scavenging Capacities

To further elucidate the differences in *R*_e^–^,UV_ between the four treatment scenarios, we calculated e_aq_^–^ formation rate () and scavenging capacity (*k*_S,*t*_^′^) as a function of UV-ARP irradiation time for each treatment scenario. For the UV/S_2_O_8_^2–^ treated ROC, higher *R*_e^–^,UV_ resulted from higher  (Figure 2A) and lower *k*_S,*t*_^′^ (Figure 2B). These changes are attributable to the decrease in DOC concentration and *A*_254_ resulting from UV/S_2_O_8_^2–^ pretreatment (Table 2). The high initial *A*_254_ limits the fraction of incoming photons that can be absorbed by the sulfite to ∼50% at the beginning of UV/SO_3_^2–^ treatment, lowering . After 1.25 h of UV/S_2_O_8_^2–^, the background *A*_254_ of the ROC is reduced from 0.964 to 0.348 cm^–1^ (Table 2). Consequently,  at the onset of UV/SO_3_^2–^ treatment increased from 3.0 × 10^–7^ to 4.0 × 10^–7^ M s^–1^ (Figure 2A). Although the DOC absorbance at 254 nm also decreases during UV/SO_3_^2–^ treatment,^45^ the abatement is markedly slower than seen with only 1.25 h of UV/S_2_O_8_^2–^. This observation is consistent with the known reactivity of electron-rich aromatic moieties with oxidizing radicals vs e_aq_^–^. For example, phenol reacts more quickly with ^•^OH ( 6.6 × 10^9^ M^–1^ s^–1^) and SO_4_^•–^ 6.06 × 10^9^ M^–1^ s^–1^) compared to e_aq_^–^ ( of 2.0 × 10^7^ M^–1^ s^–1^).^46^

The prominent impact of DOC on *k*_S,*t*_^′^ is observed between 7 and 12 h of UV/SO_3_^2–^ treatment. Due to the large contribution of nitrate to the total *k*_S,*t*_^′^ (*k*_nitrate,0_^′^ ∼ 10^7^ s^–1^*vs k*_DOC,0_^′^∼ 10^6^ s^–1^), nitrate scavenges most of the e_aq_^–^ for the first ∼7 h (Figure 2C). Once nitrate and its reduction product, nitrite, are fully degraded, DOC is the next primary e_aq_^–^ scavenger. A 7-fold reduction in *k*_S,*t*_^′^ was observed at 12 h, and a 3-fold reduction in *k*_S,*t*_^′^ was observed at 24 h for UV/S_2_O_8_^2–^ treated ROC relative to no preoxidation (Figure 2B). This 7-fold reduction is in good agreement with the 5-fold difference in *k*_S,*t*_^′^ predicted using the DOC concentrations in Table 2 (∼50 vs ∼10 mg_C_^–1^ L^–1^).

These results indicate that DOC plays a significant role in hindering the UV/SO_3_^2–^ treatment of PFAS in ROC through a combination of light shielding and scavenging of e_aq_^–^. Other concentrated waste streams, such as ion exchange still bottoms, may contain even higher DOC concentrations.^16^ Preoxidation via UV/S_2_O_8_^2–^, UV/H_2_O_2_, ozone, or other suitable oxidants will abate the DOC concentration and light absorbance, thereby increasing *R*_e^–^,UV_, and decreasing the irradiation time (i.e., energy cost) required in subsequent UV/SO_3_^2–^ treatment. Destruction of oxyanion contaminants produced during oxidation can be achieved in subsequent UV/SO_3_^2–^ treatment (see Section 3.6).

### 3.5 Relative Removal of PFOS, PFBS, PFOA, and PFBA

In the UV/S_2_O_8_^2–^ + UV/SO_3_^2–^ spikes treatment, all PFAS except PFBS were degraded below method detection limits between 12 and 24 h during UV/SO_3_^2–^ treatment (Figure 1C). The relative reactivity of PFAS followed the order PFOA ≈ PFBA ≈ PFOS ≫ PFBS. The marked difference between PFOS and PFBS is consistent with the chain length-dependent degradation of PFSAs reported in prior literature.^23,36^ The similar reactivity of PFOS, PFOA, and PFBA in the pH 12 UV/SO_3_^2–^ system is corroborated by a previous report demonstrating greater rates of parent compound attenuation and defluorination % of PFCAs and PFOS at pH 12 relative to pH 9.5.^44^ In contrast, PFCAs tend to be more reactive than PFSAs at pH 9.5.^36^

PFAS destruction occurred during UV/SO_3_^2–^ treatment only after nitrate and nitrite had been mostly degraded, starting at ∼4 h (Figures 1C and 2C). Limited PFAS destruction prior to nitrate consumption was also observed in the UV/SO_3_^2–^ treatment without preoxidation (Figure S17). However, due to the 5-fold higher *R*_e^–^,UV_ afforded by preoxidation, 90% defluorination was achieved at 24 h, whereas it took 48 h to reach only 70% defluorination without UV/S_2_O_8_^2–^ pretreatment.

The fact that fluoride production exceeded the concentration calculated from the added 25 μM PFAS and their stoichiometries is puzzling, especially because the ∼5 mg L^–1^ of F^–^ ion initially in the ROC was subtracted from the defluorination % and PFBS was degraded only to ∼50% of its initial concentration. We confirmed fluoride concentration measurements in a separate control experiment using both ion chromatography and a fluoride ion selective electrode, ruling out an instrumental artifact (Figure S19). A total oxidizable precursor (TOP) assay was also performed to evaluate whether defluorination of the PFCA precursors could be responsible. Although the concentration of PFCA increased during the TOPs assay (Σ[PFCA]_before_ and Σ[PFCA]_after_ were 1.5 and 2.2 μg L^–1^, respectively), the contribution of F^–^ coming from these precursors (∼μg L^–1^) is far less than the concentration by which the fluorine mass balance is exceeded (∼ mg L^–1^). Another possible source of F^–^ ion is fluorine-containing pharmaceuticals and personal care products, which could be expected to be elevated in the ROC. Reductive defluorination of C–F bonds has been shown to occur in such compounds.^47,48^ Future work quantifying (non-PFAS) fluorinated organic compounds in the ROC is needed to evaluate this possibility.

Interestingly, the PFAS concentrations increased during the first 2 h of UV/SO_3_^2–^ treatment after oxidation with UV/S_2_O_8_^2–^ (Figure 1C). [PFOA], [PFBA], and [PFBS] all increased by approximately 18%, while [PFOS] increased by 38%. This increase in the concentration was not observed in the UV/SO_3_^2–^ experiment without UV/S_2_O_8_^2–^ (Figure S17). Although speculative, one possible explanation is that bubbles formed during UV/S_2_O_8_^2–^ preoxidation enhanced PFAS concentrations at the air–water interface near the sampling port of the reactor.

### 3.6 Reductive Degradation of Nitrate, Bromate, and Chlorate

A drawback of UV/S_2_O_8_^2–^ pretreatment is the conversion of ammonia to nitrate, bromide to bromate, and chloride to chlorate (see the insets of Figure 2C,D). Chlorate was already present in the ROC (Table 2), but its concentration increased during UV/S_2_O_8_^2–^ preoxidation, both under conditions in which ^•^OH and SO_4_^•–^ were formed. In comparison, bromate formation was much higher when SO_4_^•–^ was the oxidant (Figures 2D and S16). Formation of nitrate, chlorate, and bromate is of concern because they are regulated contaminants and scavenge e_aq_^–^. Subsequent UV/SO_3_^2–^ treatment resulted in abatement of these oxyanions within the same time scale as PFAS destruction: nitrate (and nitrite) within ∼7 h, bromate in <4 h, and chlorate within 48 h (Figure 2C,D). Thus, UV/S_2_O_8_^2–^ pretreatment permits faster degradation of PFAS during subsequent UV/SO_3_^2–^ reductive, while simultaneously degrading undesired UV/S_2_O_8_^2–^ byproducts. Importantly, oxyanion formation could also be expected to occur in other pretreatment approaches that utilize oxidation.

### 3.7 pH Impact on Carbonate Speciation and Subsequent e_aq_^–^ Scavenging

Preliminary UV/SO_3_^2–^ experiments conducted at pH 9.5 in ROC led to lower defluorination (<30% at 24 h) than at pH 12, which is consistent with prior literature showing a marked increase in the degradation rate of both PFCAs and PFSAs with increasing pH. To differentiate between potential causes of this enhancement–scavenging by carbonate species vs hydroxide-mediated chemistry of intermediates, we used MCAA to quantify e_aq_^–^ scavenging in the UV/SO_3_^2–^ system at pH 10 and pH 12 in the presence of 10 mM total carbonate in ultrapure water. PFOA and PFOS were employed as target contaminants in these experiments.

**Figure 3:**
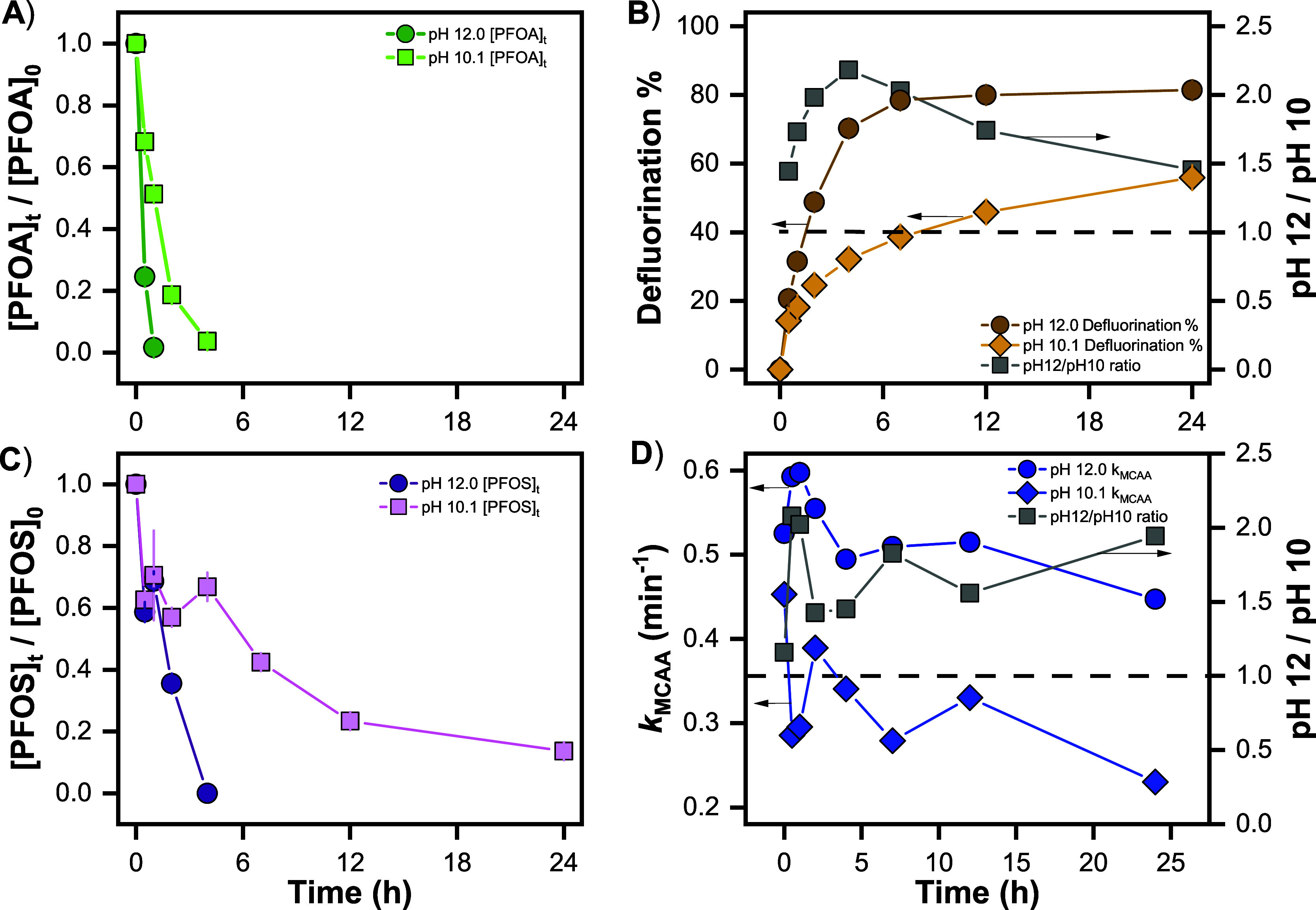
Impact of pH on UV/SO_3_^2–^ of (A) PFOA, (B) PFOS, (C) defluorination %, and (D) first-order rate constant of e_aq_^–^-mediated MCAA degradation. Gray squares in (B,D) refer to the pH 12 to pH 10 ratio (scale on right *y*-axis). Markers represent the mean of duplicate measurements, and error bars represent the range between the duplicates (some error bars are within markers). Experimental conditions: 10 W low-pressure Hg lamp, pH_0_ = 10 or 12, 20 °C, N_2(g)_ bubbling, 9.5 mM [sulfite]_0_, [MCAA]_0_ = 50 μM, [PFOS]_0_ = 25.0 μM (13.5 mg L^–1^), [PFOA]_0_ = 25.0 μM (10.9 mg L^–1^), and [C_T,CO3_]_0_ = 10.0 mM in ultrapure water.

PFOA and PFOS degraded faster at pH 12 in the UV/SO_3_^2–^ system compared to pH 10 (Figure 3A and 3C). The defluorination % was also greater at pH 12, with the enhancement ranging from ∼1.5 to 2 (Figure 3B, right *y*-axis). If this enhancement were due solely to the differences in e_aq_^–^ scavenging, then it should be possible to measure the enhancement with MCAA. Assuming the rate of e_aq_^–^ formation from sulfite photolysis is unchanged between pH 10 and 12, the ratio of first order rate constants for loss of MCAA can be shown to be equal to  (see Text S3.3). Under this assumption, the ratio of MCAA first order rate constants (*k*_MCAA_) at pH 12 to 10 is proportional to the additional e_aq_^–^ scavenging at pH 10. This ratio ranged between 1.5 and 2, indicating that the e_aq_^–^ scavenging capacity at pH 10 is up to 2-fold higher than at pH 12. The similar 2-fold enhancement in defluorination % and *k*_MCAA_ at pH 12 relative to pH 10 provides strong evidence that an increase in [e_aq_^–^], likely due to decreased e_aq_^–^ scavenging, plays an important role in the elevated PFAS destruction rates at pH 12. Reduced e_aq_^–^ scavenging may be especially important for continued destruction of polyfluorinated intermediates, which are degraded more slowly in the UV/SO_3_^2–^ system than perfluorinated compounds.^36^

A final piece of evidence supporting the role of carbonate species in e_aq_^–^ scavenging in the UV/SO_3_^2–^ system at pH 10 is the production of formate at pH 10, which was not observed at pH 12 (Figure S11). Production of formate is consistent with the reduction of CO_2(aq)_ by e_aq_^–^ (eqs 1 and 2)^46^
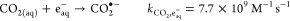
1
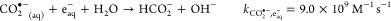
2

Although CO_2(aq)_ is a relatively small fraction of total carbonate species (*C*_T,CO_3__) at pH 10 (α_0_ = 1.5 × 10^–4^), it is still an important contributor to e_aq_^–^ scavenging due to the high C_T,CO_3__ in the system (i.e., 10 mM) and the high reactivity of H_2_CO_3_*, which is mostly CO_2(aq)_.^32^

## 4 Conclusions

Concentrated waste streams derived from membrane filtration and ion exchange regeneration are a promising target for high-energy PFAS destruction technologies such as UV-ARP. However, there is a lack of detailed knowledge regarding the role of co-occurring water matrix constituents on the effectiveness of UV-ARP in these waste streams.

This study is among the first to evaluate PFAS destruction by using UV-ARP in an authentic concentrated waste stream. Pretreatment of the ROC with UV/S_2_O_8_^2–^ led to a 3-fold enhancement in defluorination % at 24 h irradiation during subsequent UV/SO_3_^2–^ treatment compared to previously optimized UV/SO_3_^2–^ conditions (i.e., pH 12) without preoxidation. This 3-fold enhancement was attributable to the greater *R*_e^–^,UV_ in preoxidized ROC, afforded by the lower degree of light screening and diminished e_aq_^–^ scavenging of DOC. Further decreases in the irradiation time could be achieved by the targeted removal of reactive e_aq_^–^ scavengers like nitrate and its reduction product, nitrite. In our system, >4 h of irradiation with a 10 W lamp is needed in the UV/SO_3_^2–^ process to degrade ambient levels of nitrate and nitrite. If this duration was shortened, for example, by coupling less energy-demanding technologies for nitrate reduction, further energy and chemical cost savings could be realized.

An important drawback to the UV/S_2_O_8_^2–^ + UV/SO_3_^2–^ treatment is the large amount of sulfate produced. For example, sulfate concentrations increased from 1600 mg L^–1^ in the ROC (prior to treatment) to 13,000 mg L^–1^ after UV/S_2_O_8_^2–^ + UV/SO_3_^2–^ treatment.

Although we demonstrate that pH 12 results in a 2-fold reduction in e_aq_^–^ scavenging relative to pH 10 in systems containing high carbonate levels, this enhancement alone is insufficient to degrade PFAS in the ROC. These results indicate that destruction of PFAS in groundwaters with low DOC concentration will be enhanced simply by adjusting pH to 12, whereas destruction of PFAS in high DOC waters will require some form of pretreatment.

## Data Availability

Data for this article are available in the main manuscript tables and figures or in the Supporting Information. Other data will be made available upon request to the authors.

## Supporting Information Available

Materials and methods, chemicals and solution preparation details, reverse osmosis concentrate water, hardness removal pretreatment, analytical methods, *R*_e-,UV_ measurement in ROC, ionic strength calculation, nitrate as *in situ* probe compound, combined mathematical and experimental approach, results and discussion, list of chemicals and standards used, PFAS quantitative SRM transitions, measured [e_aq_^-^]_0_ values, MCAA kinetics, OCWD ROC water quality measurements, OCWD groundwater replenishment system, precipitate formation, plot of nitrate molar absorption coefficient, nitrate concentration, UV/sulfite and UV/IAA system sensitizer optimization, IAA and MCAA control experiments, MCAA degradation, spiking IAA, maximum [e_aq_^-^]_t_ determination, plot of [nitrate] versus time, formate formation, visible colorimetric change, absorbance spectra of OCWD ROC, photochemical treatment of PFAS, and relationship between fluoride ion concentration and ion chromatography (PDF)
