# Supplementary material for: Destruction of Per- and Polyfluoroalkyl Substances in Reverse Osmosis Concentrate Using UV-Advanced Reduction Processes
Source: ACS ES T Water. 2024 Oct 28;4(11):4818–27. doi: 10.1021/acsestwater.4c00458 (PMC11555674; doi:10.1021/acsestwater.4c00458)
Supplement: Supplementary file 1 — ew4c00458_si_001.pdf [file ew4c00458_si_001.pdf]

Supporting Information for

Destruction of Per- and polyfluoroalkyl Substances in Reverse Osmosis Concentrate using UV-Advanced Reduction Processes

Benjamin D. Fennell,[a] Shawnee Chavez,[a] and Garrett McKay[a]**

[a]Zachry Department of Civil & Environmental Engineering Texas A&M University, College Station, TX 77845

Corresponding authors:
Benjamin D. Fennell
Email: benjamin.fennell@utk.edu
Phone: 865.974.7597

Garrett McKay
Email: gmckay@tamu.edu
Phone: 979.458.6540

Number of Pages:	32
Number of Sections:	5
Number of Tables:	7
Number of Figures:	19

Table of Contents

S 1 Materials and Methods 4
S 1.1 Chemicals and solution preparation details. 4
S 1.2 Reverse Osmosis Concentrate Water 7
S 1.3 Hardness Removal Pretreatment. 7
S 1.4 Analytical Methods. 8
S 1.4.1 Water quality parameters: 8
S 1.4.2 Ion chromatography 8
S 1.4.3 Sulfite measurement 8
S 1.4.4 IAA measurement 9
S 1.4.5 PFAS measurement 9
S 1.4.6 Actinometry 11
S 1.4.7 Total oxidizable precursor assay 11
S 2 Calculations 11
S 2.1 $R_{e-,UV}$ Measurement in ROC. 11
S 2.2 Ionic strength calculation for ROC and ultra-pure water experiments. 12
S 2.3 Nitrate as *in situ* probe compound for $R_{e-,UV}$ determination. 12
S 2.4 Combined mathematical and experimental approach to finding maximum $[e_{aq}^-]_t$ in different UV/sensitizer systems 14
S 3 Text S3. Results and Discussion 17
S 3.1 Generation of e_{aq}^- by UV/IAA. 17
S 3.1.1 MCAA Control Experiments. 17
S 3.1.2 e_{aq}^- Generated by IAA Byproduct. 18
S 3.1.3 Spiking IAA to Maintain High $[e_{aq}^-]_t$. 19
S 3.2 Spiking chemical sensitizer at later points during UV-ARP treatment. 20
S 3.3 Bicarbonate e_{aq}^- scavenging additional discussion. 22
S 3.3.1 Derivation of the additional scavenging capacity at pH 10 versus pH 12. 22
S 4 Additional Supplementary Figures and Tables. 24
S 5 References 35

List of Supplementary Tables

Table S1. List of chemicals used in this study. 4
Table S2. List of standards used in this study. 6
Table S3. PFAS Quantitative SRM transitions. 11
Table S4. Measured $[e_{aq}^{-}]_0$ values in ultra-pure water and OCWD ROC using sulfite. 17
Table S5. Measured $[e_{aq}^{-}]_0$ values in ultra-pure water and OCWD ROC using IAA...17
Table S6. MCAA kinetics in the UV/IAA system. 19
Table S7. OCWD ROC water quality measurements for the three following experimental conditions: 1) UV-ARP/sulfite, 2) UV-ARP/IAA, or 3) UV-ARP/sulfite with UV-AOP pretreatment. 31

List of Supplementary Figures

Figure S1. OCWD Groundwater Replenishment System. 7
Figure S2. Precipitate formation in OCWD ROC with pH increase. 8
Figure S3. Plot of nitrate molar absorption coefficient ($\varepsilon_{nitrate}$) versus wavelength. 13
Figure S4. Nitrate concentration during 30 min direct photolysis experiment. 13
Figure S5. UV/sulfite and UV/IAA system sensitizer optimization in A) ultra-pure water and B) OCWD ROC. 16
Figure S6. IAA and MCAA control experiments. 18
Figure S7. MCAA degradation by e_{aq}^{-} generated by IAA byproduct. 19
Figure S8. Spiking IAA to maintain a high $[e_{aq}^{-}]_t$. 20
Figure S9. Maximum $[e_{aq}^{-}]_t$ determination for sulfite spikes at 12 h and 24 h in OCWD ROC. 21
Figure S10. Plot of [nitrate] versus time in a 0.4 mM UV/IAA experiment in OCWD ROC. 21
Figure S11. Formate formation in ultra-pure water with 10.0 mM carbonate buffer (pH 10.1 and 12.0). 22
Figure S12. Visible colorimetric change in OCWD ROC after 48 h of treatment in A) UV-ARP/sulfite and B) UV-ARP/IAA. 24
Figure S13. Absorbance spectra of OCWD ROC with and without cation exchange pretreatment. 24
Figure S14. Photochemical treatment of PFAS spiked into ROC under the following four experimental conditions: 1) UV/IAA, 2) UV/SO_3^{2-}, 3) UV/$S_2O_8^{2-}$ + UV/SO_3^{2-}, and 4) UV/$S_2O_8^{2-}$ + UV/SO_3^{2-} spikes. 25
Figure S15. Photochemical treatment of OCWD ROC under UV-ARP/sulfite spikes with UV-AOP pretreatment experimental conditions. 26
Figure S16. Photochemical treatment of OCWD ROC under UV-ARP/sulfite with UV-AOP pretreatment experimental conditions. 27
Figure S17. Photochemical treatment of OCWD ROC under UV-ARP/sulfite experimental conditions. 28
Figure S18. Photochemical treatment of OCWD ROC under UV-ARP/IAA experimental conditions. 29
Figure S19. Relationship between fluoride ion concentration measured by ion-sensitive electrode and ion chromatography (IC). 30

S 1 Text S1: Materials and Methods

S 1.1 Chemicals and solution preparation details.

All chemicals (purchased from Sigma Aldrich or VWR) and standards (purchased from Wellington Laboratories LLC and Inorganic Ventures) utilized in this study are listed in **Table S1** and **Table S2**.

Table S1. List of chemicals used in this study.

Chemical	Cas #	Purity
Ammonium acetate	127-09-3	>99%
Ammonium chloride	12125-02-9	≥99.5%
Ammonium hydroxide (28-30% NH_3)	1336-21-6	ACS reagent
Boric acid	10043-35-3	99.97%
5,5'-dithiobis(2-nitrobenzoic acid)	69-78-3	99%
Heptadecafluorooctanesulfonic acid potassium salt (PFOS)	2795-39-3	≥98%
Heptafluorobutyric acid (PFBA, 98%,)	375-22-4	≥97.5%
Hydrochloric acid (37%)	7647-01-0	ACS reagent
Indole-3-acetic acid	87-51-4	98%
Methanesulfonic acid	75-75-2	≥99.0%
Methanol	67-56-1	≥99.8% ACS
Nitric acid (70%)	7697-37-2	≥99.999%
Nonafluorobutane-1-sulfonic acid (PFBS, 97%)	375-73-5	ISO 9001
Perchloric acid (70%)	7601-90-3	ISO 9001
Potassium chloride	744-40-7	>99%
Potassium persulfate	7727-21-1	≥99%
Potassium phosphate monobasic anhydrous	7778-77-0	99%
Potassium phosphate dibasic	7758-11-4	98%
Sodium bicarbonate	144-55-8	>99.7%
Sodium bromate	7789-38-0	≥ 99%
Sodium chloroacetate	3926-62-3	98%
Sodium fluoride	7681-49-4	>99%
Sodium hydroxide (solid pellets)	1310-73-2	≥97%
Sodium iodate	7681-55-2	≥99%

Sodium nitrate	7631-99-4	≥99%
Sodium nitrite	7632-00-0	≥97%
Sodium perfluorooctanoate (PFOA)	335-95-5	97%
Sodium sulfite	7757-83-7	≥98%
Sulfuric acid (95-98 %)	7664-93-9	ACS reagent
Uridine	58-96-8	99%

Table S2. List of standards used in this study.

Chemical	Catalog or Lot #	Purity	Use Description
Perfluoro-n($^{13}C_8$)octanoic acid (M8-PFOA)	M8PFOA0522 (Lot Number)	>98%	PFOA SPE cartridge extraction efficiency
Sodium perfluoro-1-($^{13}C_8$)octanesulfonate (M8-PFOS)	M8PFOS0722 (Lot Number)	>98%	PFOS SPE cartridge extraction efficiency
Perfluoro-n(2,3,4-$^{13}C_8$)butanoic acid (M3-PFBA)	M3PFBA0822 (Lot Number)	>98%	PFBA SPE cartridge extraction efficiency
Sodium perfluoro-1-(2,3,4-$^{13}C_3$)butanesulfonate (M3-PFBS)	M3PFBS1122 (Lot Number)	>98%	PFBS SPE cartridge extraction efficiency
Buffer, precision reference standard, pH 4.000±0.002 at 25ºC (RICCA)	1502-16 (Catalog Number)	ISO 17025	pH meter calibration
Buffer, precision reference standard, pH 7.000±0.002 at 25ºC (RICCA)	1552-16 (Catalog Number)	ISO 17025	pH meter calibration
Buffer, precision reference standard, pH 10.000±0.002 at 25ºC (RICCA)	1602-16 (Catalog Number)	ISO 17025	pH meter calibration
Traceable conductivity calibration standard (VWR)	89030-260 (Catalog Number)	ISO 17025	Conductivity instrument calibration
1000 ppm 7 anion calibration standard solution (Inorganic Ventures)	IV-STOCK-59-125ML (Catalog Number)	ISO 17034	Ion chromatography calibration
Inorganic cation calibration standard solution (Inorganic Ventures)	IC-SCS1-125ML (Catalog Number)	ISO 17034	Ion chromatography calibration

All solutions were prepared by dissolving chemical salts into ultra-pure water (≥ 18.20 MΩ cm) and stored at 4^{0}C with the exception of per- and polyfluoroalkyl substances (PFAS) solutions and sodium sulfite solutions. Stock PFAS solutions (1 mM) were prepared by dissolving either chemical salts or concentrated liquid solutions into ultra-pure water, stirring (400 rpm) and heating (150) on a VWR Hotplate-Stirrer until dissolved, and then stored at room temperature. Wellington Laboratories LLC surrogate standards were used during PFAS solid phase extraction (SPE) as discussed in S 1.4.5. A working stock PFAS surrogate solution (1,500 ng mL^{-1}) was created by diluting the Wellington Laboratories LLC surrogate standard(s) (50,000 ng mL^{-1}) in HPLC grade

methanol prior to SPE. Sodium sulfite solutions were prepared by dissolving sodium sulfite salt in anerobic ultra-pure water. Anerobic conditions were created by bubbling nitrogen gas in ultra-pure water for at least 45 minutes prior to adding sodium sulfite salt and dissolving, sealing in a 40 mL amber glass vial with polypropylene open-top cap, and then bubbling nitrogen gas an additional 30 minutes to remove any oxygen present in the headspace of the glass vial. Sodium sulfite solutions were stored at room temperature and remade weekly.

S 1.2 Reverse Osmosis Concentrate Water

ROC was collected from the Orange County Water District Advanced Water Purification Facility. A schematic of this system and the and sample collection point can be found in

Figure S1. RO was shipped from OCWD to Texas A&M and, upon receipt, was filtered with muffled (500 ºC, 4 h) and pre-rinsed 1.6 µm Whatman glass microfiber filters (GF/A) and stored at 4^0 C. Water quality parameters such as anion and cation analysis, dissolved organic carbon (DOC), alkalinity, pH, specific conductance, and absorbance at 254 nm (A_{254}) were then measured

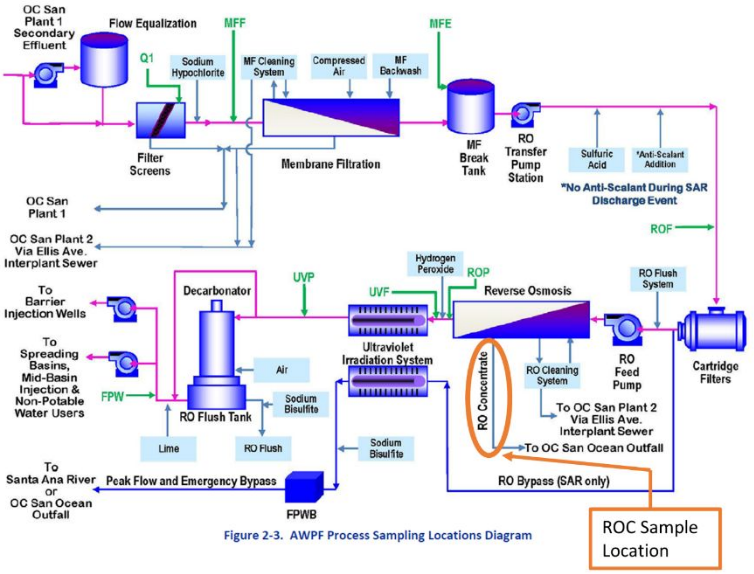

for the ROC (Table 1, main manuscript, and S 2). Native PFAS in the ROC were also quantified (S 1.4.5)

Figure S1. OCWD Groundwater Replenishment System.

S 1.3 Hardness Removal Pretreatment.

In order to conduct UV-ARP experiments, pH of the OCWD ROC was required to be raised

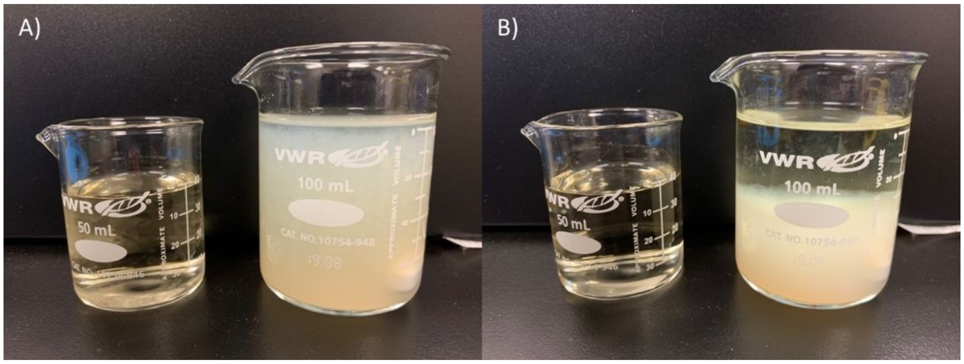

from 7.8 to 9.5-12.0. Preliminary pH adjustments with NaOH in the OCWD ROC revealed that formation of white precipitates occurred when pH was raised above 9.0 (**Figure S2A**). These precipitates could be settled within 30 min (**Figure S2B**). Reaction of the white precipitates with nitric acid produced white foaming bubbles similar to reactions between bicarbonate and acetic acid. It is hypothesized that pH increases within OCWD ROC produced calcium carbonate or other similar precipitates due to the high concentration of calcium (1,199 mg L^{-1} as $CaCO_3$) and bicarbonate (alkalinity = 1,352 mg L^{-1} as $CaCO_3$).

Figure S2. Precipitate formation in OCWD ROC with pH increase. OCWD ROC prior to pH adjustment is on left side of A) and B). Right beakers in A) and B) show OCWD ROC adjusted to pH 9.8 with NaOH after 0 min (A) and 30 min (B) of settling.

S 1.4 Analytical Methods.

S 1.4.1 Water quality parameters:

Water quality parameters were measured for experiments conducted in ROC and ultra-pure water. ROC was filtered prior to analysis with muffled (500 ºC, 4 h) and pre-rinsed 1.6 µm Whatman glass microfiber filters (GF/A). DOC quantification was conducted by Hazen Huffman Laboratories in Golden, Colorado after samples were adjusted to pH $\leq$ 2 using trace metal grade nitric acid (70%). Standard Method 2320 was used to calculate total alkalinity by titrating to pH 4.3 using 0.02 N sulfuric acid. pH was measured using a ThermoFisher Scientific Orion Versa Star Pro combined with either an Orion Triode pH probe (9107BN) or Orion Ross semi-micro electrode pH probe (8115BNUWP). The pH meter was calibrated prior to use with a standard three-point calibration curve. Absorbance spectra, including A_{254}, was measured on an Agilent Cary-100 UV-vis spectrophotometer with a 1 cm path length quartz cuvette. Specific conductance was measured on a VWR Traceable Portable Conductivity Meter after performing a standard 4-point calibration curve.

S 1.4.2 Ion chromatography

Anion and cation concentrations were quantified on a Dionex Integrion and Aquion ion chromatography system (ThermoFisher Scientific). Anion and cation analysis, with the exception of sulfite, was conducted on a ThermoFisher Scientific Dionex Integrion and Aquion ion chromatography system. The Dionex Integrion ion chromatography system (anion analysis) was equipped with a conductivity detector, a Dionex IonPac AS19 analytical column (4x250 mm), a Dionex IonPac AG19 (4x50 mm) guard column, and a Dionex ADRS 600 (4 mm) suppressor. For all ROC samples, the Dionex Integrion column operated at 30 ^{0}C with a 10 mM KOH isocratic eluent at 1.0 mL min^{-1} flow rate and a 25-mA suppressor current. All other water samples were analyzed at a 20 mM KOH isocratic eluent at a 1.0 mL min^{-1} flow rate and a 50-mA suppressor current. The Dionex Aquion ion chromatography system (cation analysis) was equipped with a conductivity detector, a Dionex IonPac CS12A analytical column (4x250 mm), a Dionex IonPac CG12A (4x50 mm) guard column, and a Dionex CDRS 600 (4 mm) suppressor. All samples were analyzed with the column operating at 30 ^{0}C with a 20 mM methanesulfonic acid isocratic eluent at a 1.0 mL min^{-1} flow rate and a 59-mA suppressor current. Some cation and anion ROC samples were diluted with ultra-pure water prior to ion chromatography measurements to fall within ion chromatography calibration curve limits.

S 1.4.3 Sulfite measurement

Sulfite concentrations were quantified using a colorimetric method developed by Humphrey *et al.*[1] To prepare samples for analysis, a 0.25 mL or 0.5 mL sample was withdrawn from our photochemical reactors and immediately diluted with ultra-pure water (40:1, 20:1, or 10:1

dilution). The ultra-pure water used here was not purged with nitrogen gas due to our previously established method.[2] 0.5 mL of the diluted sulfite sample was then added to a solution containing 4.0 mL of 100 mM phosphate buffer (pH 7) and 0.5 mL of 2.0 mM 5,5'-dithiobis(2-nitrobenzoic acid) (DTNB). For samples with pH ≥ 11, 0.01 mL of 1.0 M HCl was also added to this solution to bring the pH below 10 for accurate sulfite quantification.[1] Reaction between sulfite and DTNB then occurred for the next 5-10 minutes before measuring the absorption of thiol at 412 nm. The reference blank for absorbance measurements included 4.45-4.48 mL of 100 mM phosphate buffer (pH 7), 0.5 mL of 2.0 mM DTNB solution, and 0.0125-0.05 mL background water (e.g., equivalent volume of either ultra-pure water or ROC used in the final solution for absorbance measurements). The Beer-Lambert Law (eq. S1) was then used to calculate the concentration of sulfite using a 14,000 M^{-1} cm^{-1} molar absorption coefficient of thiol at 412 nm (ε_{412}) and a 1 cm cuvette path length (*l*).

$$A_{412} = \varepsilon_{412} l [sulfite] \quad \text{eq. S1}$$

S 1.4.4 IAA measurement

A ThermoFisher Scientific Dionex Ultimate 3000 high performance liquid chromatography (HPLC) system was utilized to quantify indole-3-acetic acid (IAA) concentrations. The HPLC system was equipped with a diode array detector and a 5 µm Supelco Ascentis Express 90 Å RP-Amide column (15 cm x 4.6 mm). IAA concentrations were analyzed with the column operating at 30 ^{0}C with a 70 % solution A (phosphoric acid at pH 3 with 10% acetonitrile) and 30% solution B (methanol) eluent at a 1.0 mL min^{-1} flow rate. IAA concentrations were determined by measuring the absorbance at a 262 nm wavelength on the diode array detector.

S 1.4.5 PFAS measurement

Following EPA Standard Method 537.1,[3] PFAS analysis involved first isolating PFAS from the background water matrix using solid phase extraction (SPE) followed by high performance liquid chromatography mass spectrometry quantification (HPLC/MS). Prior to SPE, samples were diluted (200:1) in ultra-pure water and spiked with our PFAS surrogate working stock (0.15 mL working stock to a total 10 mL volume). The PFAS surrogate working stock solution contained isotopically labeled standards for PFAS of interest (**Table S2**). The SPE and LC-MS/MS procedures are discussed in detail below.

A 16-port HyperSep Glass Block Vacuum Manifold (ThermoFisher Scientific) was prepared with Oasis WAX 1cc cartridge, 30 mg, 30 µm (Waters) for SPE. Under low vacuum pressure (≤ 2 psi), up to 8 total Oasis WAX cartridges were conditioned three consecutive times using 1 mL of 0.1% NH_4OH in methanol followed by three consecutive times using 1 mL of 100% methanol. 1 mL of sample was then immediately added onto the Oasis WAX cartridge followed by 1 mL of 25 mM ammonium acetate to wash the cartridge. All cartridges were dried under low vacuum pressure (≤ 2 psi) for 4-5 minutes. The vacuum manifold was then turned off and cartridges were moved to the back of the vacuum manifold. Under low vacuum pressure (≤ 2 psi), 1 mL of 0.1% NH_4OH in methanol was added to elute PFAS off the cartridges into disposable, borosilicate glass collection tubes (VWR). The vacuum was then turned off and all elution tubes were covered with parafilm before transferring the final product into Leap PAL Parts L-MARK vials with inserts and PTFE/silicone caps. Lower limits of quantitation via this method (0.25 µg L^{-1}) are based on the lowest concentration used in the calibration curve (0.25 ng mL^{-1}), the volume of sample loaded onto the SPE cartridge (1 or 5 mL), and the volume of 0.1 % NH_4OH used to elute the cartridge (1 mL).

PFAS concentrations were quantified on a triple quadrupole mass spectrometer (Altis, ThermoFisher Scientific, Waltham, MA) coupled to a binary pump HPLC (Vanquish,

ThermoFisher Scientific). Mass spectrometer parameters were optimized for the PFAS under direct infusion at 5 μL min^{-1} to identify the SRM transitions (precursor/product fragment ion pair) with the highest intensity (**Table S3**). Samples were kept at 4°C on an autosampler before 10 μL were injected onto the instrument. A Hypersil Gold 5 μm, 50 x 3 mm column (ThermoFisher Scientific) at 30°C was used for chromatographic separation. A 9.5-minute gradient method was used with a solvent composed of 0.1% formic acid solution in water and a 0.1% formic acid solution in acetonitrile at a 0.6 mL min^{-1} flow rate. TraceFinder 3.3 (ThermoFisher Scientific) was used for sample analysis and quantification. Final PFAS concentrations were normalized to the specific isotopically labeled PFAS surrogate concentration contained in each sample.

Table S3. PFAS Quantitative SRM transitions.

Compound	Polarity	Precursor (m/z)	Product (m/z)	Linear Range
PFOS	Negative	499.000	79.917	0.25 ng/ml to 100 ng/ml
PFOA	Negative	413.1	368.9	0.25 ng/ml to 100 ng/ml
PFBS	Negative	299.1	79.9	0.25 ng/ml to 100 ng/ml
PFBA	Negative	213.1	168.9	0.25 ng/ml to 100 ng/ml
PFHxA	Negative	313.1	268.9	0.25 ng/ml to 100 ng/ml
6:2 FTS	Negative	427.1	406.9	0.25 ng/ml to 100 ng/ml
PFPeA	Negative	263.0	218.845	0.25 ng/ml to 100 ng/ml
M8-PFOS	Negative	506.9	79.9	0.25 ng/ml to 100 ng/ml
M8-PFOA	Negative	420.9	375.9	0.25 ng/ml to 100 ng/ml
M3-PFBS	Negative	301.9	79.9	0.25 ng/ml to 100 ng/ml
M3-PFBA	Negative	215.9	171.9	0.25 ng/ml to 100 ng/ml

S 1.4.6 Actinometry

The UV irradiance (I_0) was determined in each photochemical reactor monthly using uridine actinometry[4] and eq. S2,

$$I_{0,254\ nm} = \frac{k_U[U]l}{1000\Phi_U(1-10^{-\varepsilon_{262}l[U]})} \quad \text{eq. S2}$$

where k_U is the reaction rate constant for the transformation of uridine by direct photolysis (s^{-1}), [U] is the concentration of uridine (M), l is the effective path length (cm), Φ_U is the quantum yield of uridine (0.020 mol Es^{-1}), and ε_{262} is the molar absorption coefficient for uridine at wavelength 262 nm (10,185 M^{-1} cm^{-1}). Uridine actinometry experiments involved irradiating 10 μM uridine in a 1.0 mM phosphate buffer (pH 7) solution and measuring the change in absorbance over a 10 min time span. Over the course of our experiments, I_0 values ranged from 0.99×10^{-9} – 1.21×10^{-8} Es cm^{-2} s^{-1}.

S 1.4.7 Total oxidizable precursor assay

The total oxidizable precursor (TOP) assay was modified from Houtz and Sedlak.[5] Two 100 mL solutions were prepared in duplicate 100 mL amber glass storage bottles. The control bottle contained ROC and 30 mM NaOH while the sample bottle contained ROC, 60 mM potassium persulfate, and 150 mM NaOH. The sample was placed in an oven pre-heated to 85 ºC for 6 h, while the control was placed on the benchtop for 6 h. After the assay, the sample was cooled to room temperature and control and sample solutions were sampled for PFAS using solid phase extraction and LC-MS/MS.

S 2 Text S2: Calculations

S 2.1 $R_{e-,UV}$ Measurement in ROC.

The fluence-normalized e_{aq}^- exposure ($R_{e-,UV}$) was measured during UV/SO_3^{2-} and UV/IAA experiments using MCAA as described previously.[2] Simultaneous measurement of $R_{e-,UV}$, water

absorbance, and e_{aq}^- sensitizer concentration permitted calculation of the e_{aq}^- formation rate ($R_{f,t}^{e_{aq}^-}$) and e_{aq}^- scavenging capacity ($k'_{S,t}$). $R_{e-,UV}$ was computed by measuring the degradation of a probe compound (PC) with a known bimolecular reaction rate constant (k_{PC,e_{aq}^-}) over time (eq. S3)

$$R_{e-,UV} = \frac{-ln\left(\frac{[PC]_t}{[PC]_0}\right)}{E_0 \times t \times k_{PC,e_{aq}^-}} = \frac{\int_0^t [e_{aq}^-]_t dt}{H} \quad \text{eq. S3}$$

where $\int_0^t [e_{aq}^-]_t dt$ is the e_{aq}^- exposure from time 0 to t, H is the UV fluence (mJ cm^{-2}), and E_0 is the average fluence rate (mJ cm^{-2} s^{-1}). Furthermore, if we assume that [e_{aq}^-] does not change during the $R_{e-,UV}$ sample time, we can multiply $R_{e-,UV}$ by E_0 to obtain [e_{aq}^-] at any time t ([e_{aq}^-]$_t$). The measured [e_{aq}^-]$_t$ along with a calculated $R_{f,t}^{e_{aq}^-}$ can then be used to compute $k'_{S,t}$ (s^{-1}) with eq. S4,

$$[e_{aq}^-]_t = \frac{R_{f,t}^{e_{aq}^-}}{k'_{S,t} + k'_{MCAA,t}} \quad \text{eq. S4}$$

where $k'_{MCAA,t}$ is the e_{aq}^- scavenging capacity of MCAA if spiked into solution. We selected a 50 µM MCAA probe compound concentration to minimize $k'_{MCAA,t}$ while also permitting quantification of kinetic degradation within detection limits. The total $k'_{S,t}$ and $k'_{MCAA,t}$ for the ROC can also be calculated as $\sum_i k_{S_i,e_{aq}^-}[S_i]_t$, where k_{S_i,e_{aq}^-} is the e_{aq}^- bimolecular rate constant of scavenger S and [S_i]$_t$ is the scavenger concentration at time t.

Nitrate was used as an *in-situ* probe compound for initial samples times (e.g., at t < 7 h), while 50 µM MCAA was used as a probe compound at t > 7 h. This was necessary because of the large e_{aq}^- scavenging capacity due to nitrate at t < 7 h, such that very high [MCAA] would need to be employed. Given the low conversion of nitrate over the kinetic timeframe (~5% over 5 minutes), $R_{e-,UV}$ at t < 7 h measured by nitrate is considered approximate (S 2.3).

S 2.2 Ionic strength calculation for ROC and ultra-pure water experiments.

Ionic strength for the ROC was calculated using the measured specific conductance (SC, µS cm^{-1}) as reported in Table 1 (main manuscript) and eq. S5.[6]

$$I = (1.3 \times 10^{-5}) \times SC \quad (\text{for } I < 0.5) \quad \text{eq. S5}$$

Eq. S6 was used to calculate the ionic strength from ions added to either the ROC or ultra-pure water,

$$I = \frac{1}{2}\sum_i M_i Z_i^2 \quad \text{eq. S6}$$

where M_i (molarity) was used to approximate the molality of the ith ion and Z_i is the integer number of charges on the ith ion.

S 2.3 Nitrate as *in situ* probe compound for $R_{e-,UV}$ determination.

Nitrate was used as an *in situ* probe compound (PC) to measure $R_{e,UV}$ at early time points (< 7 h) in the UV-ARP treatment of the OCWD ROC. MCAA could not be utilized at these early time points as nitrate scavenged nearly all the e_{aq}^-, leaving the [MCCA] unchanged. Even though it has been demonstrated that nitrate reacts quickly with e_{aq}^- in UV-ARP treatment ($k_{nitrate,e_{aq}^-}$ = 9.7×10^9),[7] nitrate may not be an ideal PC to quantify $R_{e,UV}$. Reasons for this include the following.

1. Nitrate undergoes direct photolysis at numerous UV wavelengths. **Figure S3** shows a plot of the molar absorption coefficient of nitrate ($\varepsilon_{nitrate}$) versus wavelength. While wavelengths below 240 nm exhibit a high $\varepsilon_{nitrate}$, the 254 nm wavelength can transform nitrate as well.

2. Nitrate is usually the dominant e_{aq}^- scavenger in surface waters. Ideal probe compounds should not contribute significantly to the total e_{aq}^- scavenging capacity of the water.
3. e_{aq}^--transformation kinetic parameters such as $R_{e,UV}$ are best calculated when the half-life of the PC is within the kinetic time period of interest. High levels of nitrate will necessitate hours of kinetic sampling to achieve its half-life.

For these reasons, $R_{e,UV}$ values measured in this report using nitrate as the PC are, at best, a good approximation of the actual value. A future sensitivity analysis should be undertaken to determine the effectiveness of nitrate as PC in relation to other e_{aq}^- PCs.

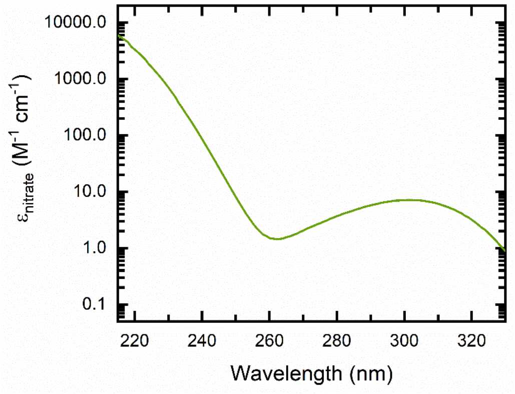

Figure S3. Plot of nitrate molar absorption coefficient (**$\varepsilon_{\text{nitrate}}$**) versus wavelength. $\varepsilon_{nitrate}$ at 254 nm wavelength was measured as 3.37 M^{-1} cm^{-1}. Sodium nitrate solutions were prepared and diluted in unbuffered ultra-pure water (final pH ranging between 5.5-5.7) for the following concentrations: 5.0 mM, 3.0 mM, 1.0 mM, 0.5 mM, 0.25 mM, and 0.10 mM. Molar absorption coefficients were derived from a linear fit of absorbance to molar concentration using absorbance values (optical density) less than 2.0.

Since nitrate can undergo direct photolysis, a preliminary experiment was conducted to see the impact of direct photolysis on our $R_{e,UV}$ approximations when using nitrate as the PC. **Figure S4** reveals that direct photolysis of nitrate has an insignificant contribution to the transformation of nitrate in UV-ARP treatment since [nitrate] was unchanged within our kinetic time span of 5.0 min.

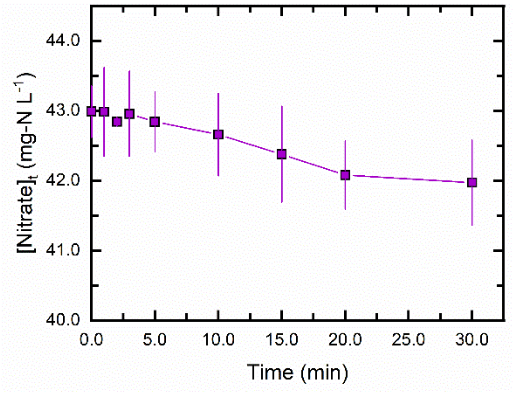

Figure S4. Nitrate concentration during 30 min direct photolysis experiment. Experiment includes the following conditions: 10 W low-pressure Hg lamp, pH_0 = 9.5, 20^{0}C, $[\text{nitrate}]_0$ =

43.0 mg-N L^{-1}, and [borate]$_0$ = 1.0 mM in ultra-pure water. Markers represent the mean of duplicate measurements and error bars represent the range between the duplicates.

S 2.4 Combined mathematical and experimental approach to finding maximum $[e_{aq}^-]_t$ in different UV/sensitizer systems

To optimize sensitizer conditions for treating PFAS in the OCWD ROC, we developed a mathematical and experimental approach to solve for the maximum ($[e_{aq}^-]_{0,max}$) irrespective of source water quality. The optimized sensitizer conditions were then used in the experimental results previously presented in Section 3.2 of the main manuscript. Using the $R_{e-,UV}$ method,[2] we were able to compare specific UV/sensitizer systems under similar reactor and lamp configurations. For this study, we selected sulfite and IAA due to their unique benefits each provide to the UV-ARP treatment of ROC and based on their use in prior studies. First, sulfite has a slow reported bimolecular rate constant for its reaction with e_{aq}^- ($k_{sulfite,e_{aq}^-}$<1.5×10^6 M^{-1} s^{-1})[7,8] and can be regenerated in soltuion after the reduction of the sulfite radical anion. Both of these reasons allow sulfite to persist throughout contaminant treatment (generally more than 24 h when using a low-pressure, Hg lamp). IAA, on the other hand, has a higher molar absoprtion coefficient ($\varepsilon_{IAA} = 2{,}576\ M^{-1}\ cm^{-1}$ vs. $\varepsilon_{sulfite} = 18.14\ M^{-1}\ cm^{-1}$)[9] of all the commonly used sensitizers,[10] permitting more UV photons to be more absorbed by IAA at an equivalent concentration of sulfite and will theoretically produce a higher $[e_{aq}^-]$. The larger ε_{IAA} value will also permit the utilization of a smaller [IAA]$_0$ than in the UV/sulfite sytem and, for an initial hypothesis, we suspected that IAA would be able to overcome the light screening effects present in the OCWD ROC ($A_{254\ nm}$ = 0.964 cm^{-1}). Additional discussion comparing the advantages and disadvantages of other chemical sensitizers can be found in our previous literature review.[9]

A plot of $[e_{aq}^-]_0$ against initial sensitizer concentration ([sens]$_0$) for both sulfite and IAA reveal the type of source water drastically impacts sensitizer selection and concentration optimization (**Figure S5**). Ultra-pure water (**Figure S5**A) has a 3-order higher $[e_{aq}^-]_0$ than OCWD ROC (**Figure S5**B) for both sensitizers. This highlights the significant $k'_{S,0}$ of the OCWD ROC. Lines in **Figure S5** represent theoretical $[e_{aq}^-]_0$ values based off eq. S6 (an expansion of eq. 3.2 in the main manuscript) whereas markers represent actual $[e_{aq}^-]_0$ values measured by the $R_{e-,UV}$ method,[2]

$$[e_{aq}^-]_0 = \frac{R_{f,0}^{e_{aq}^-}}{k'_{S,0}} = \left(\frac{\Phi_{sens,e_{aq}^-}\frac{I_0}{l}\left(1-10^{-(\varepsilon_{Sens}[sens]_t+\alpha_0)l}\right)}{k'_{S,0}+k'_{sens,0}+k'_{MCAA,0}}\right)\left(\frac{\varepsilon_{sens}[sens]_0}{\varepsilon_{sens}[sens]_0+\alpha_0}\right) \quad \text{eq. S7}$$

where Φ_{sens,e_{aq}^-} is the e_{aq}^- quantum yield of the sensitizer ($\Phi_{sulfite,e_{aq}^-} = 0.116$ Mol Es^{-1} and $\Phi_{IAA,e_{aq}^-} = 0.140$ Mol Es^{-1}),[10] I_0 is the photon irradiance (mmol photons cm^{-2} s^{-1}), ε_{sens} is the sensitizer's molar absorption coefficient (M^{-1} cm^{-1}), $[sens]_0$ is the sensitizer's initial concentration (M), α_0 is the absorption coefficient of the background water matrix at 254 nm (cm^{-1}), l is the path length (2.23 cm), and $k'_{S,0}$, $k'_{sens,0}$, $k'_{MCAA,0}$ (s^{-1}) are the initial e_{aq}^- scavenging capacities of the source water, sensitizer, and MCAA, respectively. $k'_{S,0}$ was assumed to be zero in ultra-pure water whereas $k'_{S,0}$ for the OCWD ROC was determined experimentally as 3.2 × 10^7 s^{-1} using the $R_{e-,UV}$ method. $k'_{sens,0}$ was calculated as the product of the bimolecular reaction rate constant for the sensitizer with e_{aq}^- (k_{sens,e_{aq}^-}, M^{-1} s^{-1}) and the sensitizer concentration (M). $k'_{MCAA,0}$ was

calculataed in a similar manner. Values of 1.5×10^{6} M^{-1} s^{-1}, 1.7×10^{8} M^{-1} s^{-1}, and 1.0×10^{9} M^{-1} s^{-1} were utilized for $k_{sulfite,e_{aq}^{-},lit}$, $k_{IAA,e_{aq}^{-},lit}$, and $k_{MCAA,e_{aq}^{-},lit}$, respectively.[7] These literature values were adjusted for ionic strength with the Brønsted-Bjerrum prior to calculating $[e_{aq}^{-}]_0$. **Figure S5** also presents the observed $k_{sens,e_{aq}^{-}}$ ($k_{sens,e_{aq}^{-},obs}$) values based off of our measured $[e_{aq}^{-}]_0$.

Interestingly, we discovered that sensitizer selection and the initial sensitizer concentration optimization highly depend on source water characteristics. For instance, in ultra-pure water (**Figure S5**A), the main e_{aq}^{-} scavenger in the system is the sensitizer itself ($k'_{sens,0}$), producing a theorical $[e_{aq}^{-}]_{0,max}$ for IAA and sulfite as 1.4×10^{-11} M and 1.0×10^{-11} M, respectively. Since IAA has a 142-fold higher ε_{sens}, this $[e_{aq}^{-}]_{0,max}$ value occurs at a 0.075 mM [IAA] whereas it takes ~ 10 mM [sulfite] to reach this $[e_{aq}^{-}]_{0,max}$. Again, these values are approximately 3 orders of magnitude larger than $[e_{aq}^{-}]_0$ for OCWD ROC (**Figure S5**B). The larger $k'_{S,0}$ value in the OCWD ROC both reduces $[e_{aq}^{-}]_{0,max}$ and shifts the optimal sensitizer concentration for both sensitizers. IAA actually produces an approximate 2-fold higher $[e_{aq}^{-}]_{0,max}$ when compared to sulfite in the OCWD ROC. In the OCWD ROC, the "ideal" $[IAA]_0$ and $[sulfite]_0$ approach 2.5 mM and 50 mM, respectively. This increase in optimal sensitizer concentration is driven not only by the increase in $k'_{S,0}$ but also by the observed UV photon screening impact. The chromophoric portion of the OCWD ROC, which is primarily attributed to DOC at a concentration ~50 mg_C L^{-1}, screens UV photons from illuminating the chemical sensitizer, directly decreasing the $R_{f,0}^{e_{aq}^{-}}$. For example, a 2.5 mM $[IAA]_0$ absorbs 87% of the total UV photons where as a 50 mM $[sulfite]_0$ absorbs only 48% of UV photons in the OCWD ROC. This screening affect by DOC was also observed in our previous study[2] where we used 10 mg_c L^{-1} SRNOM in ultra-pure water. Taken together, these observations indicate that the optimal sensitizer concentration and sensitizer selection are highly dependent on background source water quality.

One last finding was uncovered when we measured the $[e_{aq}^{-}]_0$ (using the $R_{e-,UV}$ method) for ultra-pure water at various sensitizer concentrations (**Figure S5**A). Intriguingly, the theorical values did not match the measured values. The reason for the discrepency is most likely attributed to the $k_{sens,e_{aq}^{-}}$ values used in the theorical calculations (eq. S6). The $k_{sulfite,e_{aq}^{-}}$ value is reported as an upper limit in the literature ($<1.5\times10^{6}$ M^{-1} s^{-1}). Furthermore, the $k_{sens,e_{aq}^{-}}$ values reported for indole in the literaure vary by 3-fold (upper end is ~7.0×10^{8} M^{-1} s^{-1}),[11] indicating that the IAA value of 1.7×10^{8} M^{-1} s^{-1} may vary as well. A fitted $k_{sens,e_{aq}^{-}}$ value was obtained for both sensitizers using the method of least squares between the measured and theoretical $[e_{aq}^{-}]_0$ values, resulting in a $k_{sulfite,e_{aq}^{-},obs}$ of 1.5×10^{5} M^{-1} s^{-1} and $k_{IAA,e_{aq}^{-},obs}$ of 6.6×10^{8} M^{-1} s^{-1}. (It is recommended that additional bimolecular rate constant experiments be conducted to confirm this finding.) The slower $k_{sulfite,e_{aq}^{-},obs}$ could help explain why sulfite lasts longer during the UV/sulfite treatment of contaminants in UV-ARP studies. Furthermore, the faster $k_{IAA,e_{aq}^{-},obs}$ value matches closely with the reported upper end $k_{IAA,e_{aq}^{-}}$ literature value. As demonstrated in S 3.1, the faster $k_{IAA,e_{aq}^{-},obs}$ is also supported by the fast degradation of IAA during typical UV-ARP treatment (e.g., 0.075 mM $[IAA]_0$ was degraded within 30 minutes in ultra-pure water). Overall, these $k_{sens,e_{aq}^{-},obs}$ values reported here are expected to have the most impact in waters with low or neglible $k'_{S,0}$ values such as ultra-pure water or groundwater.

The mathematical model presented in eq. S6 and validated by the $R_{e-,UV}$ method provides a quick tool to help select an appropriate sensitizer and optimal initial sensitizer concentration in

any source water. To deploy this mathematical model approach, the following parameters must be known:

1) $k'_{S,0}$ and $A_{254\ nm}$ of the source water,
2) photochemical characteristics of sensitizer (i.e., $\Phi_{sens,\ e_{aq}^{-}}$, ε_{sens}, $k_{sens,e_{aq}^{-}}$), and
3) reactor configuration (i.e., I_0 of the lamp(s) and the reactor path length, l).

Furthermore, this mathematical model can be used to optimize sensitizer spikes at later time points during treatment. For example, we determined it was most effective to spike 25.6 mM [sulfite] at 12 h and 14.6 mM [sulfite] at 24 h to treat PFAS in the OCWD ROC. Future research should evaluate additional sensitizers such as NTA or iodide to maximize $[e_{aq}^{-}]_t$ for PFAS treatment in UV-ARP treatment applications since these sensitizers have been utilized in other PFAS studies.[12,13] Expanding the mathematical model for sensitizers such as NTA and iodide will require the quantifiation of the $\Phi_{sens,\ e_{aq}^{-}}$ and ε_{sens} for these sensitizers.

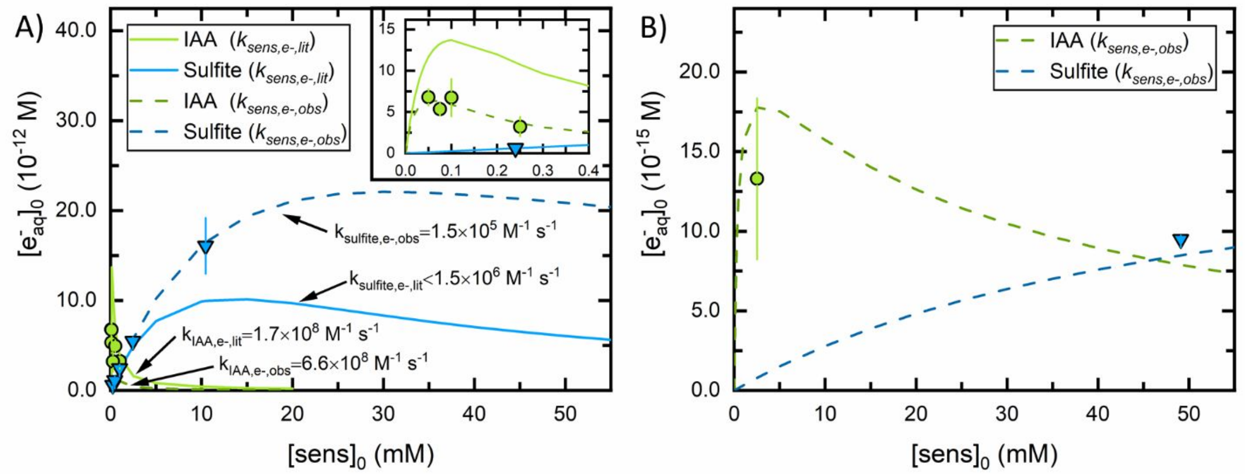

Figure S5. UV/sulfite and UV/IAA system sensitizer optimization in A) ultra-pure water and B) OCWD ROC. Markers represent measured values using the $\boldsymbol{R_{e-,UV}}$ method while lines represent theoretical values based on either literature reported $\boldsymbol{k_{sens,e_{aq}^{-}}}$ values ($k_{sens,e-,lit}$) or observed $\boldsymbol{k_{sens,e_{aq}^{-}}}$ values ($k_{sens,e-,obs}$). A literaure value of 1.5×10^6 M^{-1} s^{-1} was utilized for sulfite and a value of 1.7×10^8 M^{-1} s^{-1} was used for IAA. Markers represent the mean of duplicate measurements and error bars represent the range between the duplicates (majority of error bars are within markers). Experiments include the following conditions: 10 W low-pressure Hg lamp, pH_0 = 9.4-11.7, 20^{0}C, $N_{2(g)}$ bubbling (ultra-pure water only), $[MCAA]_0$ = 20 or 50 μM (ultra-pure water only), $[PFOS]_0$ = 25.0 μM (13.5 mg L^{-1}) and $[PFOA]_0$ = 25.0 μM (10.9 mg L^{-1}) and $[PFBS]_0$ = 25.0 μM (7.50 mg L^{-1}) and $[PFBA]_0$ = 25.0 μM (5.35 mg L^{-1}) (OCWD ROC only), and [borate] = 1.0 mM (ultra-pure water only). PFOS, PFOA, PFBS, and PFBA were spiked into the OCWD ROC at time 0.

Table S4. Measured $[e_{aq}^-]_0$ values in ultra-pure water and OCWD ROC using sulfite.

$[sulfite]_0$ [a] (mM)	$[e_{aq}^-]_0$ in ultra-pure water (M)	$[e_{aq}^-]_0$ in OCWD ROC (M)
0.24	$6.0\pm0.1\times10^{-13}$	n.r.
0.45	$1.1\pm0.06\times10^{-12}$	n.r.
0.99	$2.4\pm0.2\times10^{-12}$	n.r.
2.43	$5.5\pm0.2\times10^{-12}$	n.r.
10.44	$1.6\pm0.3\times10^{-11}$	n.r.
49.09	n.r.	$9.5\pm0.03\times10^{-15}$

[a] Experiments include the following conditions: 10 W low-pressure Hg lamp, pH_0 = 9.4-11.7, 20^{0}C, $N_{2(g)}$ bubbling (ultra-pure water only), $[MCAA]_0$ = 20.0-50.0 μM, $[PFOS]_0$ = 25.0 μM (13.5 mg L^{-1}) and $[PFOA]_0$ = 25.0 μM (10.9 mg L^{-1}) and $[PFBS]_0$ = 25.0 μM (7.50 mg L^{-1}) and $[PFBA]_0$ = 25.0 μM (5.35 mg L^{-1}) (OCWD ROC only), and [borate] = 1.0 mM (ultra-pure water only). n.r. stands for not reported.

Table S5. Measured $[e_{aq}^-]_0$ values in ultra-pure water and OCWD ROC using IAA.

$[IAA]_0$ [a] (mM)	$[e_{aq}^-]_0$ in ultra-pure water (M)	$[e_{aq}^-]_0$ in OCWD ROC (M)
0.05	$6.8\pm1.0\times10^{-12}$	n.r.
0.075	$5.3\pm0.5\times10^{-12}$	n.r.
0.1	$6.7\pm2.0\times10^{-12}$	n.r.
1	$3.3\pm0.9\times10^{-12}$	n.r.
0.5	$4.9\pm0.07\times10^{-12}$	n.r.
0.25	$3.2\pm1.0\times10^{-12}$	n.r.
2.5	n.r.	$1.3\pm0.5\times10^{-14}$

[a] Experiments include the following conditions: 10 W low-pressure Hg lamp, pH_0 = 9.4-11.7, 20^{0}C, $N_{2(g)}$ bubbling (ultra-pure water only), $[MCAA]_0$ = 20.0-50.0 μM, $[PFOS]_0$ = 25.0 μM (13.5 mg L^{-1}) and $[PFOA]_0$ = 25.0 μM (10.9 mg L^{-1}) and $[PFBS]_0$ = 25.0 μM (7.50 mg L^{-1}) and $[PFBA]_0$ = 25.0 μM (5.35 mg L^{-1}) (OCWD ROC only), and [borate] = 1.0 mM (ultra-pure water only). n.r. stands for not reported.

S 3 Text S3: Results and Discussion

S 3.1 Generation of e_{aq}^- by UV/IAA.

To determine the optimal [IAA] for contaminant degradation in the UV/IAA system, we performed several initial experiments. The discussion below presents results from experiments designed to test MCAA selective reaction with e_{aq}^-, generation of e_{aq}^- after IAA degradation, and optimizing $[e_{aq}^-]_t$ by spiking IAA throughout treatment.

S 3.1.1 MCAA Control Experiments.

Several control experiments were performed to confirm that MCAA reacts selectively with e_{aq}^- in the UV/IAA system (**Figure S6**). Under UV irradiation, 20.0 μM $[MCAA]_0$ degrades within 10 min in the presence of 0.075 mM $[IAA]_0$. We observed no change in [MCAA], however, with our dark control experiment. Furthermore, in the presence of 40.4 mg-N mL^{-1}

$[nitrate]_0$ in the OCWD ROC, we observed no change in [MCAA] during UV/IAA treatment, indicating that the e_{aq}^- generated in the UV/IAA system was completely scavenged by nitrate. Lastly, MCAA does not undergo direct photolysis at 254 nm. Taken together, these results indicate that MCAA is selective for reaction with e_{aq}^- in the UV/IAA system.

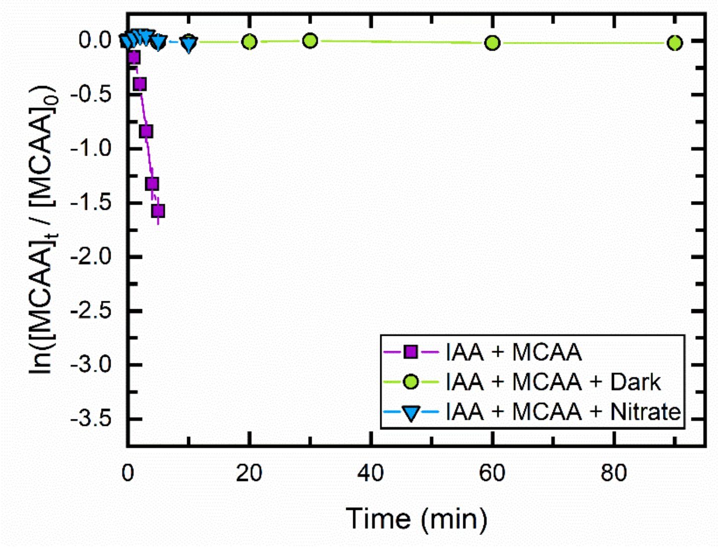

Figure S6. IAA and MCAA control experiments. IAA + MCAA and IAA + MCAA + Dark experiments include the following conditions: 10 W low-pressure Hg lamp, pH_0 = 9.3-9.5, 20^{0}C, $[IAA]_0$ = 0.075 mM, $[MCAA]_0$ = 20.0 µM, and $[borate]_0$ = 1.0 mM in ultra-pure water. IAA + MCAA + Nitrate experiment was conducted in the OCWD ROC under the following experimental conditions: pH_0 = 8.2, 20^{0}C, $[IAA]_0$ = 0.4 mM, and $[MCAA]_0$ = 20.0 µM. Markers represent the mean of duplicate measurements and error bars represent the range between the duplicates.

S 3.1.2 e_{aq}^- Generated by IAA Byproduct.

Interestingly, we discovered that MCAA continues to degrade after IAA is completely consumed in the UV/IAA system. For example, 0.1 mM $[IAA]_0$ was degraded to 0.04 mM [IAA] within 30 minutes of irradiation. We then performed a similar 4 h experiment with 0.075 mM $[IAA]_0$ but decided to spike 20.0 µM $[MCAA]_0$ into the reactors 0, 0.5 1.0, 2.0, and 4.0 h. **Figure S7** reveals that MCAA continues to degrade up to 4.0 h even though IAA has completely degraded. These results suggest that IAA may be degrading into a byproduct capable of producing e_{aq}^-. To test if the IAA byproducts are producing reactive species other than e_{aq}^-, we performed another experiment with 0.075 mM $[IAA]_0$ and 20.0 µM $[MCAA]_0$. After 30 minutes of irradiation, we turned off the UV lamps and observed no transformation of [MCAA] during this timeframe (**Figure S7**). These experimental results indicate that a byproduct from the reaction of IAA and e_{aq}^- produces e_{aq}^- which is capable of degrading contaminants like MCAA.

Table S6. MCAA kinetics in the UV/IAA system.

Time [a] (min)	$\ln\left(\frac{[MCAA]_t}{[MCAA]_0}\right)$	[IAA] (μM)
0	0	112.8±1.8
1	-0.18±0.08	n.r.
2	-0.41±0.18	n.r.
3	-0.67±0.28	n.r.
5	-1.24±0.46	n.r.
10	-2.78±0.69	n.r.
20	n.r.	n.r.
30	n.r.	41.3±5.5

[a] Experiment includes the following conditions: 10 W low-pressure Hg lamp, pH_0 = 9.5, 20°C, $[IAA]_0$ = 0.1 mM, $[MCAA]_0$ = 20.0 μM, and $[borate]_0$ = 1.0 mM in ultra-pure water. n.r. stands for not reported.

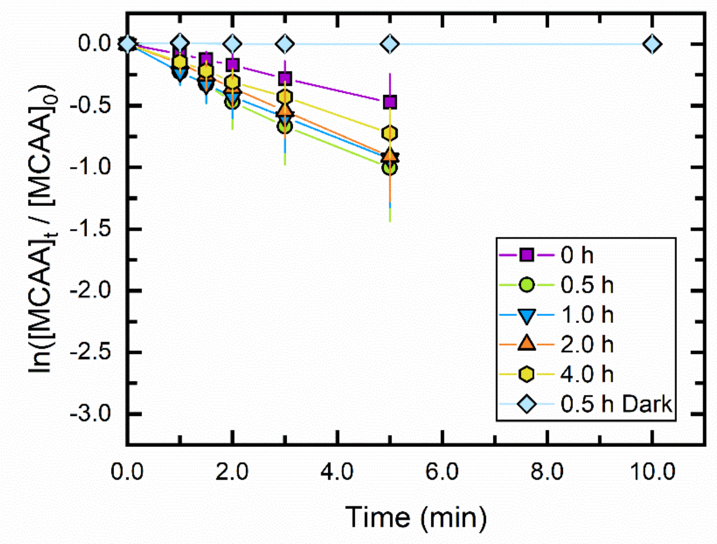

Figure S7. MCAA degradation by e_{aq}^- generated by IAA byproduct. Experiment includes the following conditions: 10 W low-pressure Hg lamp, pH_0 = 9.7, 20°C, $[IAA]_0$ = 0.075 mM, $[MCAA]_0$ = 20.0 μM spikes, and $[borate]_0$ = 1.0 mM in ultra-pure water. Markers represent the mean of duplicate measurements and error bars represent the range between the duplicates.

S 3.1.3 Spiking IAA to Maintain High $[e_{aq}^-]_t$.

Although e_{aq}^- was produced well after IAA was consumed, MCAA transformation kinetics degreased after 4 h, indicating that PFAS treatment over a 24 h timeframe could be challenging. To maintain a high $[e_{aq}^-]_t$ throughout treatment, an experiment was conducted in which 0.075 mM $[IAA]_0$ was spiked into the system at 0 h and 1 h. The results shown in **Figure S8** demonstrate that MCAA degradation rates were not only maintained but actually increased from the initial 4 h experiment (**Figure S7**).

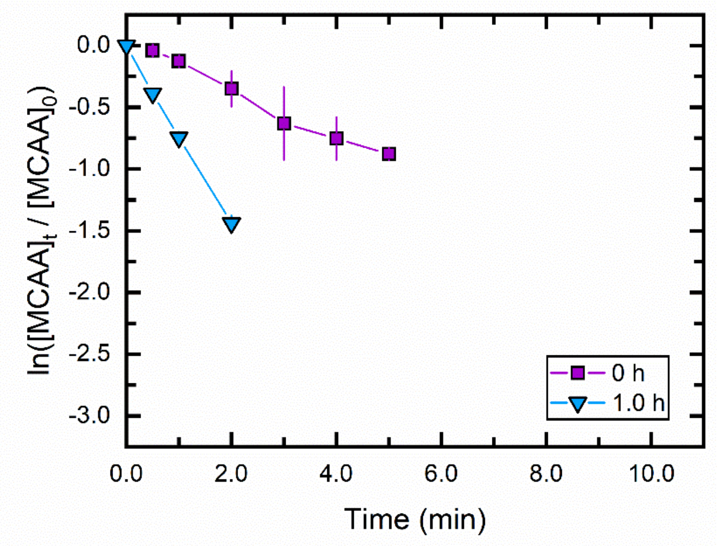

Figure S8. Spiking IAA to maintain a high $[e_{aq}^{-}]_t$. Experiment includes the following conditions: 10 W low-pressure Hg lamp, $pH_0 = 9.3$, 20°C, $[IAA]_0 = 0.075$ mM, $[MCAA]_0 = 20.0$ µM spikes, and $[borate]_0 = 1.0$ mM in ultra-pure water. Markers represent the mean of duplicate measurements and error bars represent the range between the duplicates.

S 3.2 Spiking chemical sensitizer at later points during UV-ARP treatment.

A previous study discovered that PFOS can be more readily degraded in the UV/sulfite system when sulfite is spiked at later time points during a 24 h experiment.[14] Using the model presented in eq. S6, we are able to determine the additional yield of $[e_{aq}^{-}]_t$ if two sensitizers of interest were spiked into the OCWD ROC throughout irradiation. We were further able to determine when these spikes were needed during treatment and at what concentration these spikes should occur. Our methodology for spiking sulfite and IAA at later time points is discussed below.

We determined that sulfite should be spiked at 12 h and 24 h in our 48 h experiment. The experimental results are presented as the UV/sulfite spikes with AOP experiment in Figure 1 (main manuscript). The time points of 12 h and 24 h were selected because these were one-fourth and one-half of the total treatment time. Initially, we determined that ~ 50 mM [sulfite] yielded the largest $[e_{aq}^{-}]_t$ while treating the OCWD ROC (**Figure S5**). The optimal sulfite concentration required to produce $[e_{aq}^{-}]_{max}$ at later times points, however, will be less than 50 mM due to the $k'_{S,t}$ of the OCWD ROC changing throughout treatment. We thus used the $k'_{S,12\ h}$ and $k'_{S,24\ h}$ measured during the UV/sulfite experiment (Figure 2, main manuscript) to determine the $[sulfite]_{12\ h}$ and $[sulfite]_{24\ h}$ spikes. The results are presented in **Figure S9**.

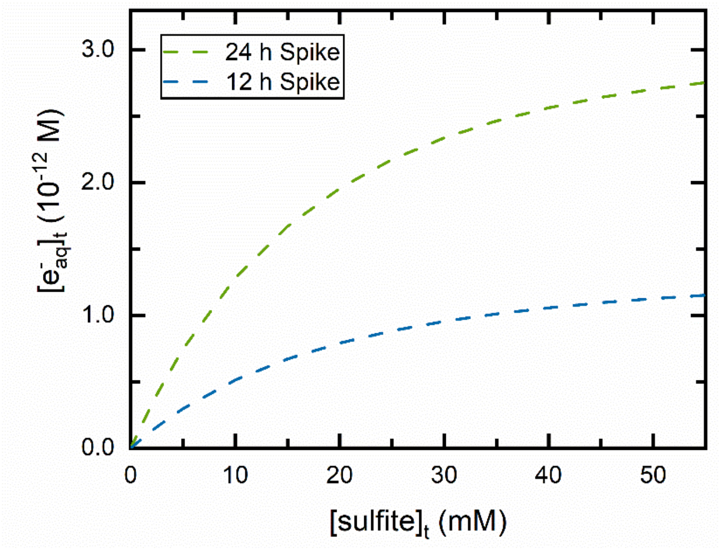

Figure S9. Maximum [e_{aq}^-]$_t$ determination for sulfite spikes at 12 h and 24 h in OCWD ROC.

Once the [sulfite]$_{12\ h}$ = 30-40 mM and [sulfite]$_{24\ h}$ = 30-40 mM from **Figure S9**, a real-time measurement was taken of the [sulfite] at 12 h and 24 h. The difference in sulfite concentration was then added to the reactors to reach ~35 mM [sulfite]$_{12\ h}$ and ~40 mM [sulfite]$_{24\ h}$, respectively.

The methodology for spiking 2.5 mM [IAA] at 12 h and 24h in our 48 h experiment (results presented in Figure 1 in the main manuscript) was slightly different from that of the sulfite spikes. Since we were not able to measure the [IAA] in OCWD ROC, we had to estimate how long the sensitizer would remain in solution and generate [e_{aq}^-]. As shown in **Figure S10**, a preliminary experiment with the OCWD ROC demonstrated that a 0.4 mM [IAA]$_0$ could significantly degrade nitrate (i.e., generate [e_{aq}^-]) for at least 2 h. We thus estimated that our 2.5 mM [IAA]$_0$ would be able to generate [e_{aq}^-] for 12 h in the OCWD ROC and decided to spike 2.5 mM [IAA] at 12 h and 24 h.

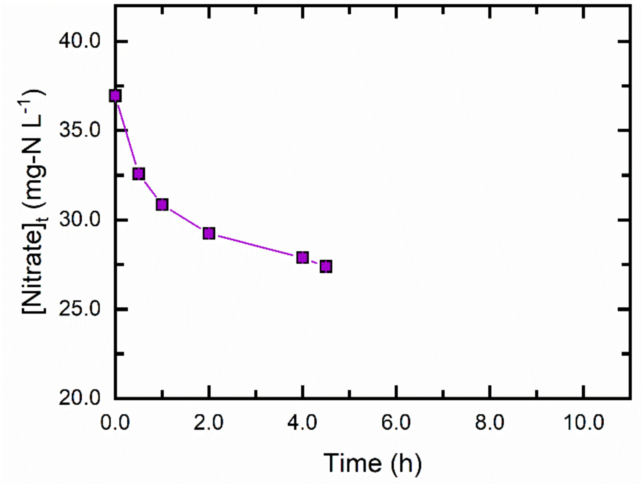

Figure S10. Plot of [nitrate] versus time in a 0.4 mM UV/IAA experiment in OCWD ROC. Experiment includes the following conditions: 10 W low-pressure Hg lamp, $pH_0 = 8.2$, 20^{0}C, [IAA]$_0$ = 0.4 mM, [MCAA]$_0$ = 50.0 µM spikes, and in OCWD ROC. Markers represent the mean of duplicate measurements and error bars represent the range between the duplicates.

S 3.3 Bicarbonate e_{aq}^- scavenging additional discussion.

As discussed in Section 3.7 in the Main Manuscript, carbonate speciation plays a major role in e_{aq}^- scavenging. The presence of bicarbonate at pH 10.1 significantly inhibited the degradation of PFAS and the formation of e_{aq}^- when compared to the predominant carbonate speciation at pH 12.0 (see Figure 3 in Main Manuscript). Interestingly, we observed the production of formate (HCO_2^-) during the UV-ARP treatment at pH 10.1 (**Figure S11**). The formation of HCO_2^- provided us a clue as the possible mechanism for reaction between e_{aq}^- and the carbonate species. We propose that the reaction of HCO_3^- and e_{aq}^- leads to the eventual product of CO_2. A final piece of evidence supporting the role of carbonate species in e_{aq}^- scavenging in the UV/SO_3^{2-} system at pH 10 is the production of formate at pH 10, but not in experiments performed at pH 12 (Figure S11). Production of formate is consistent with reduction of $CO_{2(aq)}$ by e_{aq}^- (eqs. S 7 and S 8)

$$CO_{2(aq)} + e_{aq}^- \rightarrow CO_2^{\bullet} \qquad k_{CO_2,e_{aq}^-} = 7.7 \times 10^9\ M^{-1}\ s^{-1} \qquad \text{eq. S7}$$

$$CO_2^{\bullet-}{}_{(aq)} + e_{aq}^- + H_2O \rightarrow HCO_2^- + OH^- \qquad k_{CO_2^-,e_{aq}^-} = 9.0 \times 10^9\ M^{-1}\ s^{-1} \qquad \text{eq. S8}$$

While $CO_{2(aq)}$ is a relatively small fraction of total carbonate species ($C_{T,CO3}$) at pH 10 (α_0 = 1.5×10^{-4}), it is still an important contributor to e_{aq}^- scavenging due to the high $C_{T,CO3}$ in the system (i.e., 10 mM) and the high reactivity of $H_2CO_3^*$, which is mostly $CO_{2(aq)}$.[15]

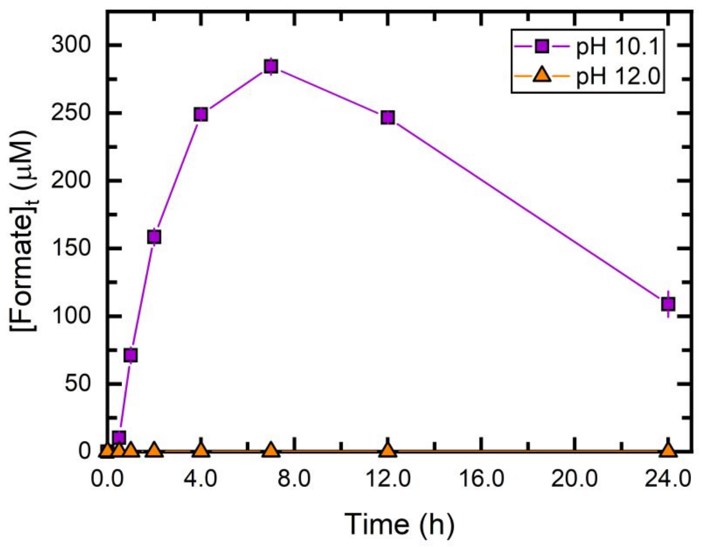

Figure S11. Formate formation in ultra-pure water with 10.0 mM carbonate buffer (pH 10.1 and 12.0). Experiments include the following conditions: 10 W low-pressure Hg lamp, pH_0 = 10.1 or 12.0, 20^{0}C, $N_{2(g)}$ bubbling, $[MCAA]_0$ = 50.0 µM spikes, $[PFOS]_0$ = 25.0 µM (13.5 mg L^{-1}), $[PFOA]_0$ = 25.0 µM (10.9 mg L^{-1}), and $[C_{T,CO3}]_0$ = 10.0 mM in ultra-pure water.

S 3.3.1 Derivation of the additional scavenging capacity at pH 10 versus pH 12.

Assuming that the rate of e_{aq}^- formation from sulfite photolysis is unchanged between pH 10 and 12, the ratio of first order rate constants for loss of MCAA (k_{MCAA}) can be shown to be equal to $1 + \left(\sum k_s[S]\right)_{pH10}/k_{MCAA}[MCAA]$. First, recognize that k_{MCAA} is equal to the product of the bimolecular rate constant and concentration of e_{aq}^-. Then, take the ratio of k_{MCAA} at pH 10 to pH 12:

$$\frac{(k_{MCAA})_{pH12}}{(k_{MCAA})_{pH10}} = \frac{\left([e_{aq}]_t \times k_{MCAA,e_{aq}^-}\right)_{pH12}}{\left([e_{aq}]_t \times k_{MCAA,e_{aq}^-}\right)_{pH10}}$$

$$= \frac{\left(R_{f,t}^{e_{aq}^-}/k'_{S,t}\right)_{pH12}}{\left(R_{f,t}^{e_{aq}^-}/k'_{S,t}\right)_{pH10}} = \frac{\left(k'_{S,t}\right)_{pH12}}{\left(k'_{S,t}\right)_{pH10}}$$

$$= \frac{k_{MCAA,e_{aq}^-}[MCAA] + \sum k_S[S]}{k_{MCAA,e_{aq}^-}[MCAA]} = 1 + \frac{\sum k_S[S]}{k_{MCAA,e_{aq}^-}[MCAA]}$$

Therefore, the ratio of k_{MCAA} at pH 12 to pH 10 provides an assessment of the extra e_{aq}^- scavenging at pH 10, which is discussed in the main manuscript to arise mostly from carbonate species.

S 4 Text S4 Additional Supplementary Figures and Tables.

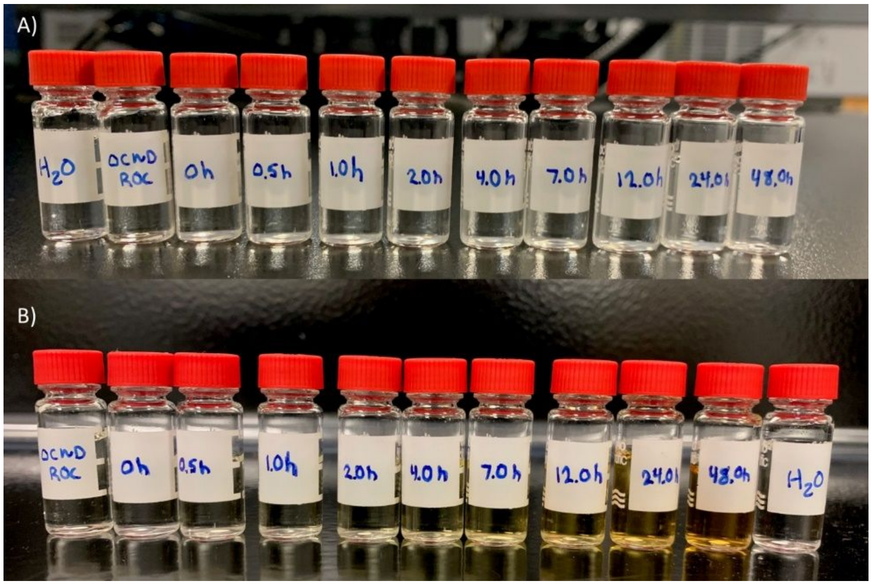

Figure S12. Visible colorimetric change in OCWD ROC after 48 h of treatment in A) UV-ARP/sulfite and B) UV-ARP/IAA. Experiments include the following conditions: 10 W low-pressure Hg lamp, pH_0 = 11.6-11.7, 20^0C, $[SO_3^{2-}]_0$ = 49.1 mM, $[IAA]_0$ = 2.5 mM, $[PFOS]_0$ = 25.0 µM (13.5 mg L^{-1}), $[PFOA]_0$ = 25.0 µM (10.9 mg L^{-1}), $[PFBS]_0$ = 25.0 µM (7.50 mg L^{-1}), $[PFBA]_0$ = 25.0 µM (5.35 mg L^{-1}), and $[MCAA]_0$ spikes = 50 µM. UV-ARP/IAA included a 2.5 mM [IAA] spike at 12 h and 24 h. PFOS, PFOA, PFBS, and PFBA were spiked into the waters at time 0. Markers represent the mean of duplicate measurements and error bars represent the range between the duplicates.

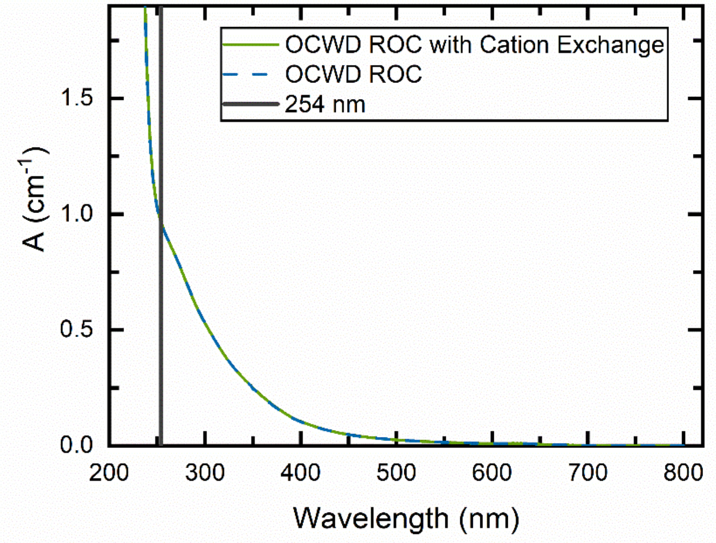

Figure S13. Absorbance spectra of OCWD ROC with and without cation exchange pretreatment.

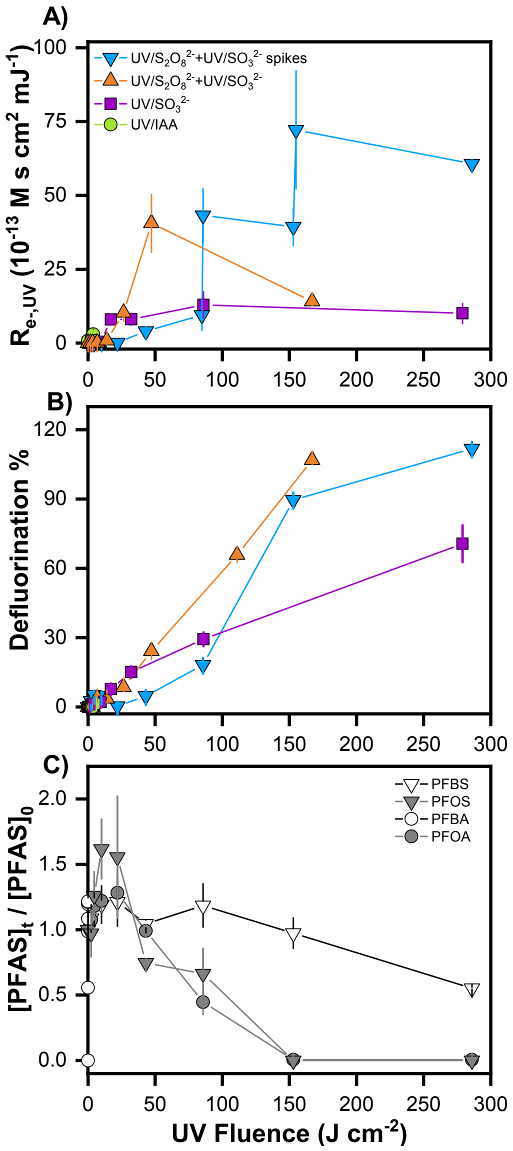

Figure S14. Photochemical treatment of PFAS spiked into ROC under the following four experimental conditions: 1) UV/IAA, 2) UV/SO_3^{2-}, 3) UV/$S_2O_8^{2-}$ + UV/SO_3^{2-}, and 4) UV/$S_2O_8^{2-}$ + UV/SO_3^{2-} spikes. $R_{e\text{-},\,UV}$ results and defluorination % for all treatment scenarios are shown in A) and B), respectively. Legend in A) applies to B). Degradation of parent PFAS from the UV/$S_2O_8^{2-}$ + UV/SO_3^{2-} spikes treatment is shown C). Markers represent the mean of duplicate measurements and error bars represent the range between the duplicates (some error bars are within markers). Experimental conditions for each of the four treatment scenarios are described in Table 1. General conditions: 10 W low-pressure Hg lamp, pH_0 = 9.5 or 12, 20^0C, $[S_2O_8^{2-}]_0$ = 25.0 mM, $[SO_3^{2-}]_0$ = 50 mM, and $[IAA]_0$ = 2.5 mM. Total fluence (F_0, J cm^{-2}) is given by $F_0 = E_0 t \times \mathrm{WF}$ where E_0 is the fluence rate (J cm^{-2} s^{-1}), t is the reaction time (s), and WF is the awter factor (WF= $(1 - 10^{-a\ell})/(2.303 \times a\ell)$.

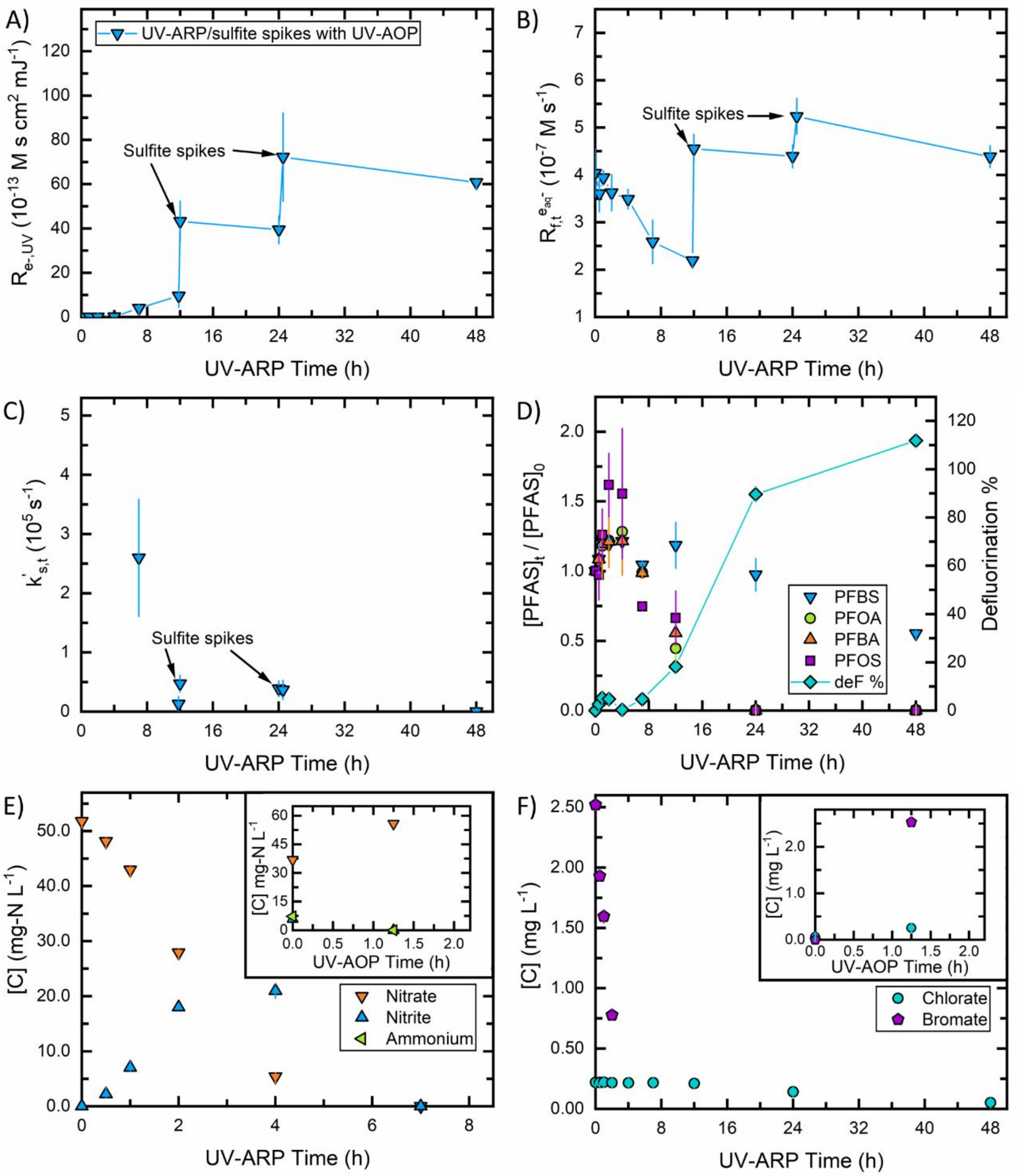

Figure S15. Photochemical treatment of OCWD ROC under UV-ARP/sulfite spikes with UV-AOP pretreatment experimental conditions. Photochemical results presented include A) $R_{e-,\,UV}$, B) rate of e_{aq}^- formation ($R_{f,t}^{e_{aq}^-}$), and C) e_{aq}^- scavenging capacity ($k'_{S,t}$). Contaminant degradations results include D) PFAS degradation and defluorination % (deF%), E) nitrate/nitrite, and F) bromate/chlorate. Markers represent the mean of duplicate measurements and error bars represent the range between the duplicates (some error bars are within markers). Experiment includes the following conditions: 10 W low-pressure Hg lamp, $pH_0 = 11.1$, 20^0C, $[SO_3^{2-}]_0 = 42.4$ mM, $[PFOS]_0 = 25.0$ μM (13.5 mg L^{-1}), $[PFOA]_0 = 25.0$ μM (10.9 mg L^{-1}), $[PFBS]_0 = 25.0$ μM (7.50 mg L^{-1}), $[PFBA]_0 = 25.0$ μM (5.35 mg L^{-1}), and $[MCAA]_0$ spikes = 50 μM at 4 h and beyond. PFOS, PFOA, PFBS, and PFBA were spiked into the waters at time 0.

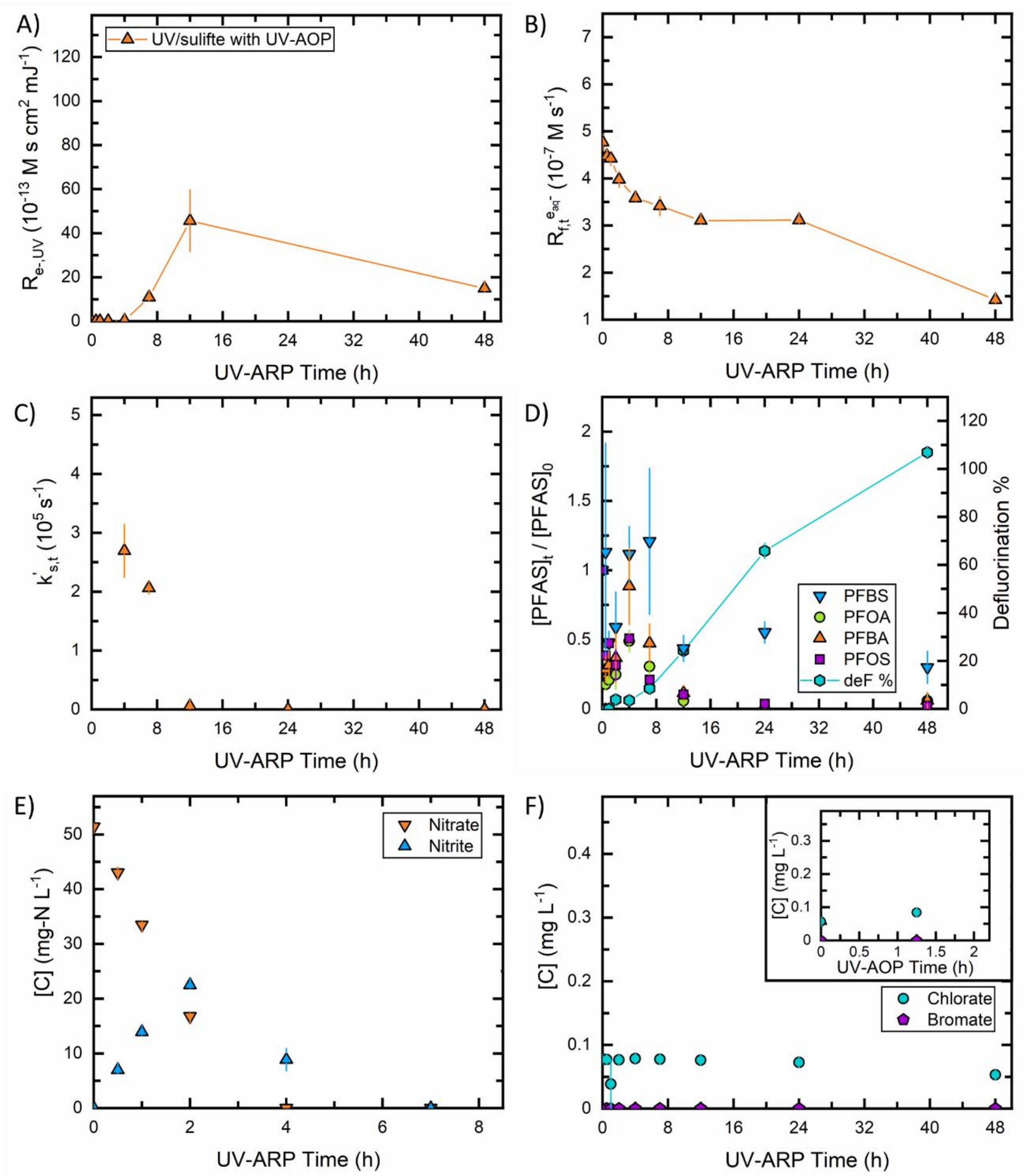

Figure S16. Photochemical treatment of OCWD ROC under UV-ARP/sulfite with UV-AOP pretreatment experimental conditions. Photochemical results presented include A) $R_{e-,\,UV}$, B) rate of e_{aq}^- formation ($R_{f,t}^{e_{aq}^-}$), and C) and e_{aq}^- scavenging capacity ($k'_{S,t}$). Contaminant degradations results include D) PFAS degradation and defluorination % (deF%), E) nitrate/nitrite, and F) bromate/chlorate. Markers represent the mean of duplicate measurements and error bars represent the range between the duplicates (some error bars are within markers). Experiment includes the following conditions: 10 W low-pressure Hg lamp, pH_0 = 11.8, 20^{0}C, $[SO_3^{2-}]_0$ = 54.0 mM, $[PFOS]_0$ = 25.0 μM (13.5 mg L^{-1}), $[PFOA]_0$ = 25.0 μM (10.9 mg L^{-1}), $[PFBS]_0$ = 25.0 μM (7.50 mg L^{-1}), $[PFBA]_0$ = 25.0 μM (5.35 mg L^{-1}), and $[MCAA]_0$ spikes = 50 μM at 4 h and beyond. PFOS, PFOA, PFBS, and PFBA were spiked into the waters at time 0.

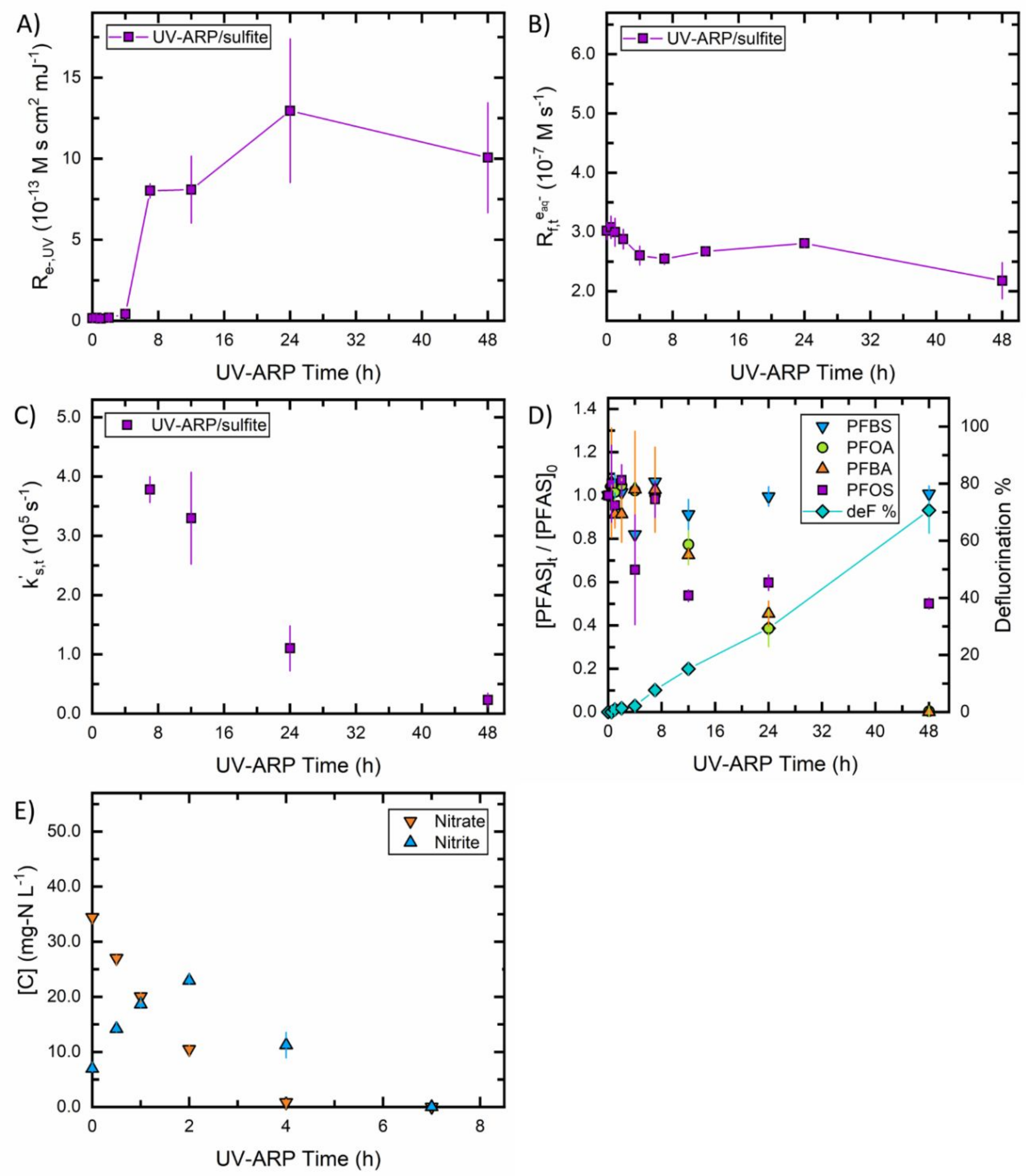

Figure S17. Photochemical treatment of OCWD ROC under UV-ARP/sulfite experimental conditions. Photochemical results presented include A) $R_{e\text{-},\,UV}$, B) rate of e_{aq}^- formation ($R_{f,t}^{e_{aq}^-}$), and C) and e_{aq}^- scavenging capacity ($k'_{S,t}$). Contaminant degradations results include D) PFAS degradation and defluorination % (deF%) and E) nitrate/nitrite degradation. Markers represent the mean of duplicate measurements and error bars represent the range between the duplicates (some error bars are within markers). Experiment includes the following conditions: 10 W low-pressure Hg lamp, $pH_0 = 11.6$, 20^0C, $[SO_3^{2-}]_0 = 49.1$ mM, $[PFOS]_0 = 25.0$ µM (13.5 mg L^{-1}), $[PFOA]_0 = 25.0$ µM (10.9 mg L^{-1}), $[PFBS]_0 = 25.0$ µM (7.50 mg L^{-1}), $[PFBA]_0 = 25.0$ µM (5.35 mg L^{-1}), and $[MCAA]_0$ spikes = 50 µM at 4 h and beyond. PFOS, PFOA, PFBS, and PFBA were spiked into the waters at time 0.

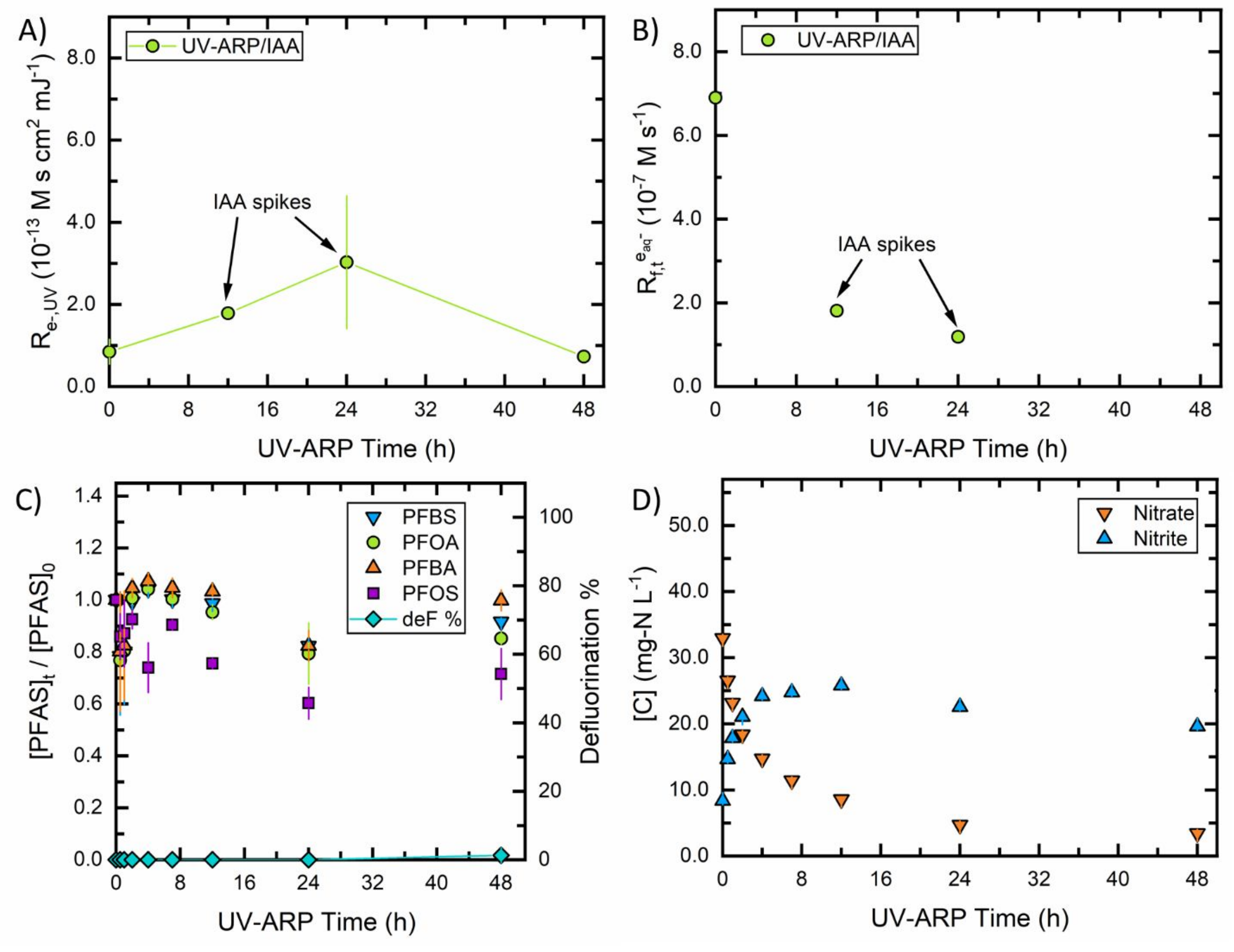

Figure S18. Photochemical treatment of OCWD ROC under UV-ARP/IAA experimental conditions. Photochemical results presented include A) $R_{e-,\,UV}$ and B) rate of e_{aq}^- formation ($R_{f,t}^{e_{aq}^-}$). Contaminant degradations results include C) PFAS degradation and defluorination % (deF%) and D) nitrate/nitrite degradation. Markers represent the mean of duplicate measurements and error bars represent the range between the duplicates (some error bars are within markers). Experiment includes the following conditions: 10 W low-pressure Hg lamp, pH_0 = 11.7, 20^{0}C, $[IAA]_0$ = 2.5 mM, $[PFOS]_0$ = 25.0 μM (13.5 mg L^{-1}), $[PFOA]_0$ = 25.0 μM (10.9 mg L^{-1}), $[PFBS]_0$ = 25.0 μM (7.50 mg L^{-1}), $[PFBA]_0$ = 25.0 μM (5.35 mg L^{-1}), and $[MCAA]_0$ spikes = 50 μM at 4 h and beyond. Experiment included a 2.5 mM [IAA] spike at 12 h and 24 h. PFOS, PFOA, PFBS, and PFBA were spiked into the waters at time 0

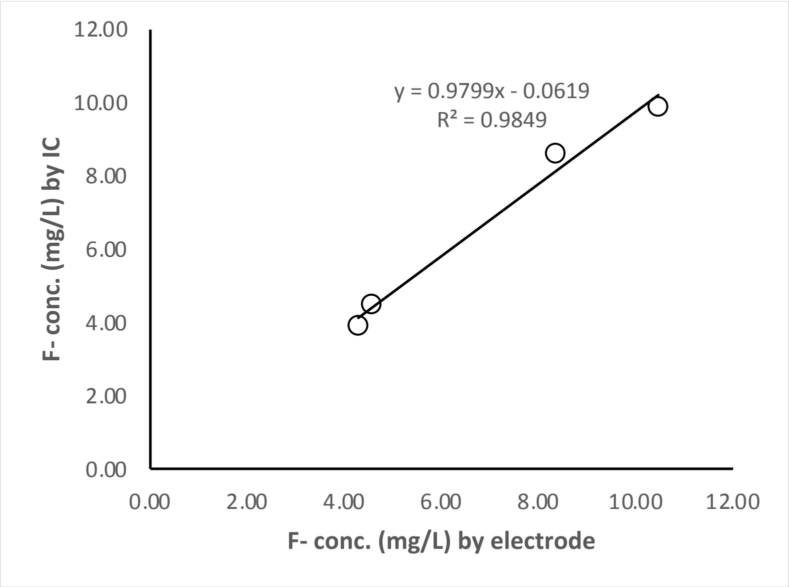

Figure S19. Relationship between fluoride ion concentration measured by ion-sensitive electrode and ion chromatography (IC).

Table S7. OCWD ROC water quality measurements for the three following experimental conditions: 1) UV-ARP/sulfite, 2) UV-ARP/IAA, or 3) UV-ARP/sulfite with UV-AOP pretreatment. Experiments include the following conditions: 10 W low-pressure Hg lamp, pH_0 = 8.2-12.0, 20^{0}C, $[S_2O_8^{2-}]_0$ = 25.0 mM, $[SO_3^{2-}]_0$ = 42.4-54.0 mM, $[IAA]_0$ = 2.5 mM, $[PFOS]_0$ = 25.0 μM (13.5 mg L^{-1}), $[PFOA]_0$ = 25.0 μM (10.9 mg L^{-1}), $[PFBS]_0$ = 25.0 μM (7.50 mg L^{-1}), $[PFBA]_0$ = 25.0 μM (5.35 mg L^{-1}), and $[MCAA]_0$ spikes = 50 μM at 4 h and beyond. UV-ARP/sulfite with UV-AOP pretreatment experiment included a 0.2 g NaOH spike at 2 h, 25.6 mM [sulfite] spike at 12 h, and 14.6 mM [sulfite] spike at 24 h. UV-ARP/IAA included a 2.5 mM [IAA] spike at 12 h and 24 h. PFOS, PFOA, PFBS, and PFBA were spiked into the waters at time 0. n.r. stands for not reported.

UV-ARP/sulfite Experiment

UV ARP Time (h)	$A_{254\ nm}$ (cm^{-1})	pH	[sulfite] (mM)	[Fluoride] [a] mg L^{-1}
0	1.660±0.022	11.6	49.1±0.01	0
0.5	1.612±0.042	11.6	48.7±0.03	0
1.0	1.624±0.061	11.7	47.7±0.38	0.21±0.04
2.0	1.573±0.028	11.7	44.4±0.19	0.31±0.08
4.0	1.545±0.025	11.9	39.4±0.42	0.49±0.06
7.0	1.437±0.012	12.1	35.9±0.29	1.75±0.22
12.0	1.196±0.005	11.9	31.4±0.21	3.44±0.19
24.0	0.798±0.011	11.9	22.3±1.07	6.69±0.68
48.0	0.392±0.021	12.0	9.60±2.20	16.1±1.82

UV-ARP/IAA

UV ARP Time (h)	$A_{254\ nm}$ [b] (cm^{-1})	pH	[IAA] [d] (mM)	[Fluoride] [a] mg L^{-1}
0	6.326±0.182	11.7	2.5	0
0.5	8.986±0.100	11.6	n.r.	0
1.0	6.472±0.720	11.6	n.r.	0
2.0	9.808±0.092	11.6	n.r.	0
4.0	15.38±0.279	11.6	n.r.	0
7.0	17.02±0.679	11.6	n.r.	0
12.0 [c]	24.10±0.668	11.5	n.r.	0
24.0	36.76±0.528	11.3	n.r.	0
48.0 [c]	48.60±1.035	11.0	n.r.	0.29±0.09

[a] Fluoride concentrations were corrected by subtracting $[fluoride]_0$ by $[fluoride]_t$; [b] Absorbance samples were diluted 20:1 prior to measurement with exception of 24 h and 28 h samples which were diluted 40:1 prior to measurement; [c] Spiked 2.5 mM [IAA]; [d] [IAA] could not be measured with HPLC due to background interference with OCWD; [e] Added 0.1 g NaOH; [f] Spiked 25.6 mM [sulfite]; [g] Spiked 14.6 mM [sulfite].

Table S7 continued.

UV-ARP/sulfite spikes with UV-AOP Pretreatment with spikes					
UV AOP Time (h)	$A_{254\ nm}$ (cm^{-1})	pH	[persulfate] (mM)	[Fluoride] [a] mg L^{-1}	[Bromide] mg L^{-1}
0	1.390±0.010	8.3	25.0	0	8.30±0.31
1.25	0.348±0.001	4.7		0.20±0.20	2.01±0.08
UV ARP Time (h)	$A_{254\ nm}$ (cm^{-1})	pH	[sulfite] (mM)	[Fluoride] [a] mg L^{-1}	[Bromide] mg L^{-1}
0	0.907±0.008	11.1	42.4±2.05	0	2.01±0.08
0.5	0.774±0.002	10.3	32.6±1.20	0.59±0.42	3.26±0.10
1.0	0.658±0.015	9.8	30.8±0.53	1.19±0.25	4.20±0.07
2.0 [e]	0.551±0.007	10.7	24.3±0.56	1.10±0.11	5.32±0.02
4.0	0.489±0.002	11.6	21.3±0.24	n.r.	7.44±0.13
7.0	0.385±0.002	11.9	13.3±1.51	1.07±0.60	7.97±0.01
11.8	0.278±0.007	11.7	9.20±0.70	n.r.	n.r.
12.0 [f]	0.641±0.003	11.7	34.8±0.14	4.15±0.68	7.04±0.13
24.0	0.531±0.019	10.8	28.6±1.45	20.4±0.73	6.75±0.03
24.5 [g]	0.699±0.019	10.8	43.2±1.20	n.r.	n.r.
48.0	0.528±0.036	10.8	28.4±2.25	25.5±0.66	6.91±0.01

UV-ARP/sulfite spikes with UV-AOP Pretreatment					
UV AOP Time (h)	$A_{254\ nm}$ (cm^{-1})	pH	[persulfate] (mM)	[Fluoride] [a] mg L^{-1}	[Bromide] mg L^{-1}
0	1.612±0.014	11.3	25.0	0	n.r.
1.25	0.668±0.105	12.0		1.62±0.92	n.r.
UV ARP Time (h)	$A_{254\ nm}$ (cm^{-1})	pH	[sulfite] (mM)	[Fluoride] [a] mg L^{-1}	[Bromide] mg L^{-1}
0	1.238±0.024	11.8	54.0±1.93	0	n.r.
0.5	1.135±0.037	11.8	46.5±0.06	0	n.r.
1.0	1.158±0.010	11.9	46.9±0.18	0	n.r.
2.0	1.136±0.006	12.2	41.4±0.63	0.89±0.10	n.r.
4.0	1.059±0.003	12.2	34.7±0.03	0.81±0.22	n.r.
7.0	0.905±0.022	12.1	28.5±0.37	1.95±0.19	n.r.
12.0	0.795±0.003	12.1	22.9±0.99	5.52±0.86	n.r.

24.0	0.699±0.014	12.0	20.5±0.03	15.01±0.75	n.r.
48.0	0.492±0.003	11.8	6.9±0.21	24.37±0.31	n.r.

[a] Fluoride concentrations were corrected by subtracting $[fluoride]_0$ by $[fluoride]_t$; [b] Absorbance samples were diluted 20:1 prior to measurement with exception of 24 h and 28 h samples which were diluted 40:1 prior to measurement; [c] Spiked 2.5 mM [IAA]; [d] [IAA] could not be measured with HPLC due to background interference with OCWD; [e] Added 0.1 g NaOH; [f] Spiked 25.6 mM [sulfite]; [g] Spiked 14.6 mM [sulfite]. n.r. stands for not reported.

S 5 References

1. Humphrey, R. E.; Ward, M. H.; Hinze, W., Spectrophotometric Determination of Sulfite with 4,4'-Dithiodipyridine and 5,5'-Dithiobis-(2-Nitrobenzoic Acid). *Analytical Chemistry* **1970,** *42* (7), 698-702.

2. Fennell, B. D.; Odorisio, A.; McKay, G., Quantifying Hydrated Electron Transformation Kinetics in UV-Advanced Reduction Processes Using the Re-,UV Method. *Environmental Science & Technology* **2022,** *56* (14), 10329-10338.

3. Method 537.1. In *Determination of Selected Per- and Polyfluorinated Alkyl Substances in Drinking Water by Solid Phase Extraction and Liquid Chromatography/Tandem Mass Spectrometry (LC/MS/MS)*, Center for Environmental Solutions & Emergency Response, Office of Research and Development, Environmental Protection Agency: Cincinnati, Ohio, 2020.

4. Jin, S.; Mofidi, A. A.; Linden, K. G., Polychromatic UV fluence measurement using chemical actinometry, biodosimetry, and mathematical techniques. *Journal of Environmental Engineering-Asce* **2006,** *132* (8), 831-841.

5. Houtz, E. F.; Sedlak, D. L., Oxidative Conversion as a Means of Detecting Precursors to Perfluoroalkyl Acids in Urban Runoff. *Environmental Science & Technology* **2012,** *46* (17), 9342-9349.

6. Brezonik, P. L.; Arnold, W. A., *Water Chemistry: An Introduction to the Chemistry of Natural and Engineered Aquatic Systems*. Oxford University Press, Inc.: 2011.

7. Buxton, G. V.; Greenstock, C. L.; Helman, W. P.; Ross, A. B., Critical Review of rate constants for reactions of hydrated electrons, hydorgen atoms, and hydroxyl radicals in Aqueous Solution. *Journal of Physical and Chemical Reference Data* **1988,** *17* (2), 513-886.

8. Maza, W. A.; Breslin, V. M.; Plymale, N. T.; DeSario, P. A.; Epshteyn, A.; Owrutsky, J. C.; Pate, B. B., Nanosecond transient absorption studies of the pH-dependent hydrated electron quenching by HSO3−. *Photochem. Photobiol. Sci.* **2019,** *18* (6), 1526-1532.

9. Fennell, B. D.; Mezyk, S. P.; McKay, G., Critical Review of UV-Advanced Reduction Processes for the Treatment of Chemical Contaminants in Water. *Acs Environ Au* **2022,** *2* (3), 178-205.

10. Fennell, B. D.; Mezyk, S. P.; McKay, G., Critical Review of UV-Advanced Reduction Processes for the Treatment of Chemical Contaminants in Water. *ACS Environmental Au* **2022,** *2* (3), 178–205.

11. Shetiya, R. S.; Rao, K. N.; Shankar, J., Determination of rate constants for the reactions of H, OH and e aq with indole-3-acetic acid and other plant hormones. *Radiation Effects* **1972,** *14* (3-4), 185-189.

12. Sun, Z.; Zhang, C.; Xing, L.; Zhou, Q.; Dong, W.; Hoffmann, M. R., UV/Nitrilotriacetic Acid Process as a Novel Strategy for Efficient Photoreductive

Degradation of Perfluorooctanesulfonate. *Environmental Science & Technology* **2018,** *52* (5), 2953-2962.

13. Liu, Z. K.; Chen, Z. H.; Gao, J. Y.; Yu, Y. C.; Men, Y. J.; Gu, C.; Liu, J. Y., Accelerated Degradation of Perfluorosulfonates and Perfluorocarboxylates by UV/Sulfite plus Iodide: Reaction Mechanisms and System Efficiencies. *Environmental Science & Technology* **2022,** *56* (6), 3699-3709.

14. Liu, Z.; Bentel, M. J.; Yu, Y.; Ren, C.; Gao, J.; Pulikkal, V. F.; Sun, M.; Men, Y.; Liu, J., Near-Quantitative Defluorination of Perfluorinated and Fluorotelomer Carboxylates and Sulfonates with Integrated Oxidation and Reduction. *Environmental Science & Technology* **2021,** *55* (10), 7052-7062.

15. Amador, C. K.; Cavalli, H.; Tenorio, R.; Tetu, H.; Higgins, C. P.; Vyas, S.; Strathmann, T. J., Influence of Carbonate Speciation on Hydrated Electron Treatment Processes. *Environmental Science & Technology* **2023**.
